# Harmonizing Peak Matching between Multidimensional NMR Spectra

**DOI:** 10.1021/acs.jcim.6c00627

**Published:** 2026-08-07

**Authors:** Anthony C. Bishop, Kyle Mimun, Weimin Tan, Taylor R. Cole, A. Joshua Wand

**Affiliations:** †Departments of Biochemistry & Biophysics and ‡Chemistry, Texas A&M University, College Station, Texas 77843, United States

## Abstract

A common task in the analysis of multidimensional NMR spectroscopy is the mapping of cross-peaks from one spectrum to another. In some situations, only a subset of coordinates of cross-peaks are to be matched, such as in comparing corresponding dimensions of triple resonance assignment spectra. In other cases, the sample is perturbed in some way, and the task is to map corresponding cross-peaks that potentially change in all dimensions. This exercise is commonly done “by hand” and is both time-consuming and subjective. It is difficult for an individual to consider all possible interpretations of deviations between two multidimensional NMR spectra. Furthermore, in regions of high degeneracy, cross-peak matchings between spectra can be ambiguous. Ideally, a mapping algorithm should reflect confidence in its matchings to indicate the reliability of proposed matchings and should do so while harmonizing all potential mappings in the spectrum. Critically, this process should be automated using criteria that are both well-defined and consistent and provide a measure of reliability. Here, we develop a novel model describing the distribution of apparent deviations between cross-peaks of two multidimensional NMR spectra due to random noise and true deviations to harmonize the cross-peak matching. Bayesian inference is employed to provide confidence estimates. The resulting algorithm (pHarmony) is tested in a variety of ways such as mapping between pairs of triple resonance spectra and between two- and three-dimensional heteronuclear NMR spectra generated by a fragment-based ligand discovery screen.

## Graphical abstract

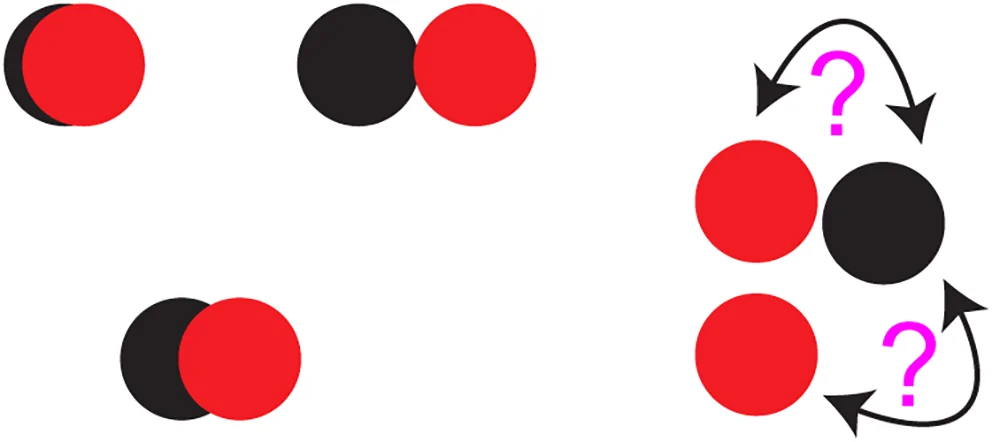

## Introduction

Analysis of multidimensional NMR spectra invariably requires the mapping of individual cross-peaks arising from a set of common spin resonances. In some cases, the mapping is a subset of nuclei such as the comparison of amide N–H correlations in, for example, HNCO and HN­(CA)­CO spectra. In other cases, the entire spin system being correlated is mapped to a corresponding cross-peak arising in a perturbed spectrum. Chemical shift perturbations can arise from changes in physical environment (e.g., temperature, pressure, ionic strength, etc.), variations between protein samples that are ostensibly the same or the addition of a ligand such as might occur in a ligand discovery campaign. Tracking the movement of cross-peaks provides valuable biophysical information on the system of interest. Often cross-peaks are identified (assigned) in a reference spectrum and “matched” to their corresponding cross-peaks in other spectra, most often as a part of a manual workflow. Should the number of spectra and number of involved cross-peaks be small, manual peak picking and matching is not a significant bottleneck. More commonly, the number and complexity of spectra to be compared, such as those encountered in a triple-resonance assignment project or an NMR based fragment-based drug discovery screening campaign, becomes unwieldy and automation of the process is desirable. Furthermore, tracking cross-peaks between spectra can be ambiguous and there is great utility in having an estimate of confidence in proposed cross-peak matches.

Though numerous automated peak picking algorithms have been proposed^1–3^ relatively few algorithms exist for the purpose of cross-peak matching, do not operate on spectra of arbitrary dimensionality or, importantly, generally do not provide estimates of confidence for the proposed matches. For example packages such as PICASSO,^4^ APET/PROPET,^5^ NvMAP,^6^ TINT^7^ and PeakWalker^8^ are designed to function only on 2D spectra. There are other algorithms that serve similar but distinct roles. CSP Analyzer^9^ is a machine learning algorithm that takes a reference and ligand perturbed peak list and classifies the liganded spectrum as being significantly perturbed or not, without providing explicit peak-wise matches. Other software packages offer a more general resonance matching capability such as ARTINA-CST,^10^ which provides a more general resonance-assignment transfer capability, but requires source chemical shift information together with peak lists from a minimal set of target spectra, including 2D HSQC and 3D NOESY-type spectra, rather than operating as a general pairwise peak list matching algorithm. Furthermore, the software packages TINT, PeakWalker and GAPT, are primarily designed and validated for the use of titration spectra and integrate the information from peak lists across multiple samples assuming linear peak trajectories, as opposed to finding matches between only two spectra. Many cross-peak matching algorithms are also designed to work on manually picked peak lists, where the peak picking was done with expert reliability and consistency and not with automated algorithms which may fail to consistently pick cross-peaks across spectra in regions of high degeneracy. To address these limitations, we have developed the cross-peak matching algorithm pHarmony, which can be used on spectra of arbitrary dimensionality, provides a meaningful confidence score for its proposed matches, and remains accurate when handling automatically picked peak lists.

## Theory

Here we outline the pHarmony algorithm which is designed to harmonize the mapping of cross-peaks of one spectrum (the reference) with the cross-peaks of another spectrum (the target). A graphical summary of the probability model employed is provided in Figure 1. The various elements of the model are described below. Some details are omitted below, but are provided in the Supporting Information.

**Figure 1:**
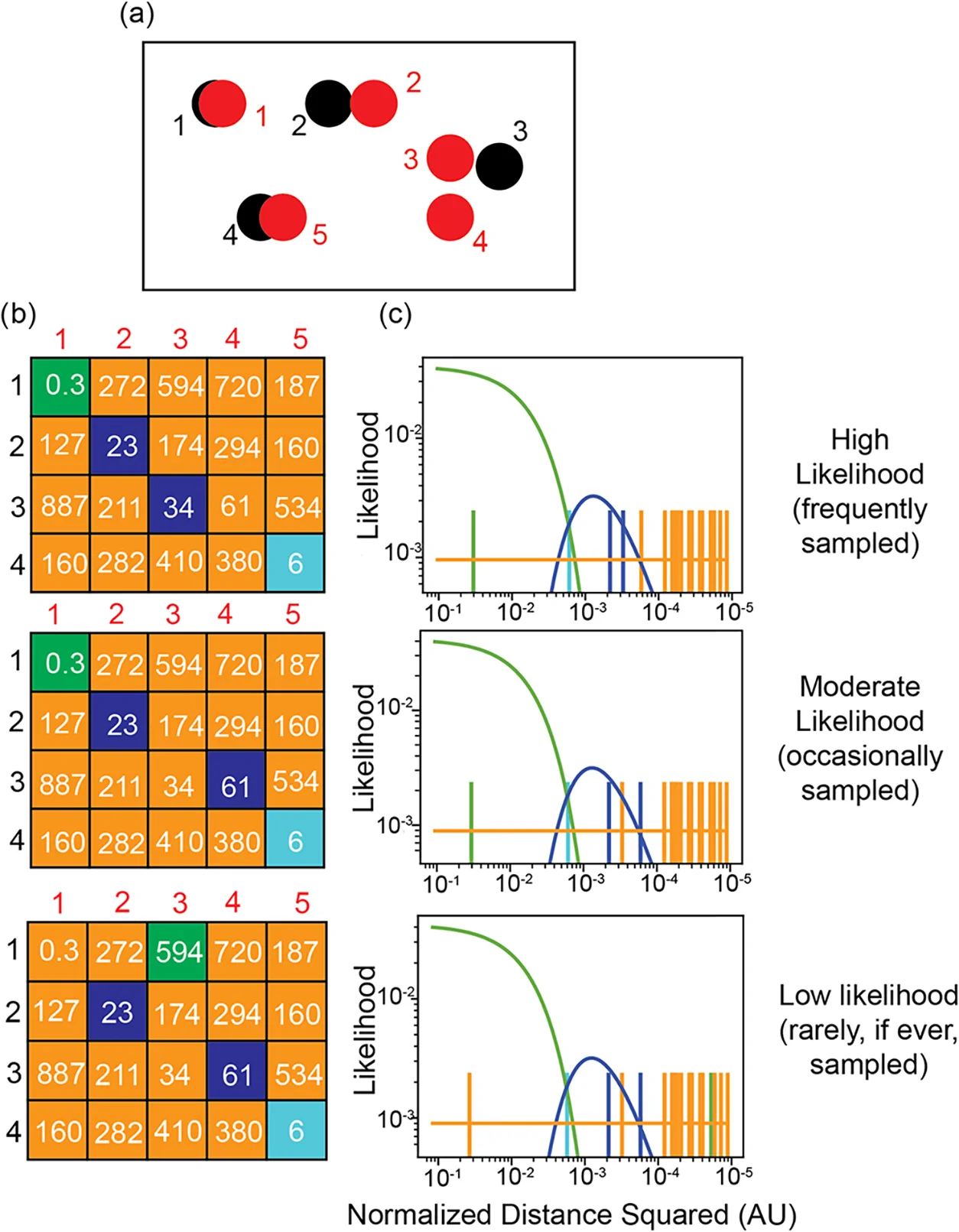
Probability model underlying the pHarmony algorithm. (Panel a) Schematic of an overlay of a reference spectrum (black) and target spectrum (red). Numbers correspond to indices in Panel b. (Panel b) Examples of sampled matching matrices with the value of the squared normalized distance between the corresponding peaks (arbitrary units) in each cell. Orange tiles correspond to cross-peak pairs that were not matched. Green, blue and cyan tiles correspond to cross-peaks that were matched with distances signifying random noise, arising from chemical shift perturbation (CSP) or with similar probability of arising from random noise or CSP, respectively. High likelihood (top), moderate likelihood (middle) and low likelihood (bottom) matching matrices are shown as examples. (Panel c) Likelihood plots corresponding to (b) as a function of squared normalized distance for each matching matrix at a fixed distribution parametrization in log–log space. Green lines correspond to a χ^2^ distribution with two degrees of freedom to model squared normalized distance arising from random noise between matching cross-peaks. Blue lines correspond to a Fréchet distribution of fixed parametrization modeling squared normalized distance arising from true CSPs between matching cross-peaks. Horizontal orange lines correspond to the constant likelihood corresponding to nonmatching cross peak pairs. Each vertical bar is a tick mark corresponding to a cell in the matching matrix placed on the horizontal axis at its value. Its color indicates the classification made (see Panel b). Distances are more likely to be classified into a given distribution according to the density of that distribution at a specific squared normalized distance. In principle, the frequency at which each matching matrix will appear is proportional to its likelihood (see Sampling the Posterior Distribution).

The problem of matching *R* reference spectrum cross-peaks to *T* target spectrum cross-peaks can be described as finding the *R* × *T* matrix *M*. Entries in *M* are either a one (1) or a zero (0) where a value of 1 at position *r, t* describes a match between the reference peak *r* and the target peak *t*. A zero indicates not a match. We constrain the problem domain to circumstances where cross-peak movement is due to either spectral noise or fast-exchange phenomena such as that which generally occurs with weak ligand binding. Thus, *M* is subject to the constraint that there can be at most a single “1” in any given row and column. This enforces exclusivity (i.e., a reference cross-peak can only match at most one target peak and vice versa). We permit *M* to encode circumstances where cross-peaks have no match (i.e., rows and columns that are filled with zeros) to account for the possibility that a cross-peak present in one spectrum has no match in the other. This provides tolerance for the situation where a peak may have disappeared for physical reasons (e.g., due to intermediate exchange broadening) as well as making it robust to errors in upstream data analysis (e.g., erroneous cross-peak picking).

Effectively, in current manual approaches, the finding of *M* leads directly to subsequent analysis. However, it is often difficult to be certain that any proposed matching matrix is equivalent to the true matching matrix *M*. For example, it is often the case that a spectral perturbation (e.g., due to ligand binding) could induce chemical shift perturbations (CSPs) in multiple cross-peaks in a crowded region. Without additional information, assignment of matches is ambiguous even for the experienced spectroscopist. The values of certain chemical shifts can themselves be informative in aiding matching.^11^ However, in circumstances where ambiguity persists, instead of only proposing a single matching matrix as the most likely *M*, it is more valuable to model the matching matrix *M* itself as a random variable and determine its Bayesian posterior probability distribution. With proper design, this posterior probability distribution would appropriately model the epistemological uncertainty of the matches and permit propagation of matching uncertainty to downstream analysis.

If a model is defined for calculating the likelihood of any possible *M* (defined as *M*_*i*_), it becomes possible to apply Bayes’ rule to calculate its posterior probability according to eq 1

1 $$P(M_{i}\;|\;D,\theta ,\phi ,\omega )=\frac{L(D\;|\;M_{i},\theta ,\phi ,\omega )P\left(M_{i}\right)}{\sum_{j}L(D\;|\;M_{j},\theta ,\phi ,\omega )P\left(M_{j}\right)}$$

*L* is a function that describes the likelihood of observed peak perturbations given a possible matching matrix *M*_*i*_ and parameters θ, ϕ, ω. The parameters are used to model the probability of observing *D*, which is a matrix of all pairwise, normalized perturbations squared, given a proposed matching matrix and are used to control the shape of the parametrized distributions within *L*. *P*(*M*_*i*_) is the prior probability of any possible *M*_*i*_. We assume that the prior distribution of *M*_*i*_ is uniform and can thus ignore its contribution to posterior probability essentially assuming that *P*(*M*_*i*_) is equal for all valid *M*_*i*_.

Explicitly calculating the partition function (i.e., the denominator of eq 1) would require calculating the likelihood of all possible *M*_*i*_. Given that the number of possible *M*_*i*_ scales combinatorially with the number of cross-peaks, an explicit calculation of the partition function is intractable for even a dozen or so cross-peaks. Furthermore, the values of the likelihood function parameters (i.e., θ, ϕ,ω) are not known *a priori* and need to be determined.

### Feature Extraction

Perhaps the most intuitive spectral feature to use to match cross-peaks between reference and target spectra is the chemical shift distance between them. The pHarmony algorithm calculates an *R* × *T* normalized distance squared matrix *D* between all possible pairs of reference and target cross-peaks. Calculation of an uncertainty-normalized distance squared between reference peak *r* and target peak *t* utilizes the eq 2

2 $$D_{\mathrm{rt}}=\sum_{k=1}^{K}\frac{{{(\delta }_{r,k}-\delta_{t,k})}^{2}}{{\sigma_{r,k}}^{2}+{\sigma_{t,k}}^{2}}$$

Where δ_*r*,*k*_ and δ_*t*,*k*_ are the chemical shifts of cross-peaks *r* and *t* respectively, along spectral dimension *k; σ*_*r*,*k*_ and *σ*_*t,k*_ are the estimated uncertainty in the position of cross-peaks *r* and *t* respectively along spectral dimension *k;* and *K* is the number of spectral dimensions. A separate uncertainty for each dimension in each spectrum is used for their respective peak uncertainties. Elements of *D* are referred to below as normalized distances squared.

### Classification and Likelihood Model

The algorithm models the problem of matching *r* reference spectrum cross-peaks to *t* target spectrum cross-peaks as an unsupervised classification problem of the normalized pairwise squared distances. The matching matrix *M* can be thought of as a classification of each element in *D*. Each element of a possible matching matrix *M*_*i*_ classifies its corresponding element within *D* to one of two possible categories: (1) the two cross-peaks defining the distance do not match or (2) the two cross-peaks defining the distance do match, subject to the previously mentioned constraints of at most one-to-one matching of reference to target cross-peaks. Of the distances that correspond to a match, we then classify these further into matching cross-peaks with a distance that arises due to random noise or due to an actual CSP (i.e., a real difference). This second layer of classification is described by matrix *C* with dimensions *R × T*. Each element *C*_*rt*_ is either a zero or a one depending on whether the corresponding entry in *D* is classified as arising due to only random noise or due to a CSP, given the corresponding cross-peaks match. Unlike *M*_*i*_ where the probability that each element is a one or a zero is highly dependent on the other elements, it is assumed that the probability that the value of any element *C*_*rt*_ is independent of all other elements in *C*. Given a pair of matched cross-peaks, the probability that the matched pair arose from a CSP or noise is independent of whether any other pair of matched cross-peaks arose from a CSP or noise. Each of these categories in which a distance *D*_*rt*_ can be placed (nonmatching pair, matching pair perturbed by noise, matching pair with a CSP), will have a defined likelihood function that is used to calculate the likelihood of an observed *D*_*rt*_ given that the corresponding peak pair is assigned to that category. The likelihood of the full matrix can then be calculated by taking the product of the likelihood of the category assigned to each distance. Figure 1 shows a cartoon example of a matching problem, three possible matching matrices of varying likelihood, and plots of the likelihood functions themselves. The likelihood functions of each category are defined below:

### Matching Cross-Peaks Perturbed by Random Noise (*M*_*rt*_ = 1, *C*_*rt*_ = 0)

For the elements *D*_*rt*_ that arise between matching cross-peaks differing in position from random noise, the positions of the corresponding cross-peaks *r* and *t* are modeled as multivariate Gaussian random variables centered at the observed chemical shifts (e.g., δ_*r*,*k*_ and δ_*t*,*k*_) with standard deviations given by the uncertainties (σ_*r*,*k*_ and σ_*t*,*k*_) for each spectral dimension *k*. In these circumstances *D*_*rt*_ is the magnitude of the difference between two normalized multivariate Gaussian random variables squared. Thus, *D*_*rt*_ can be modeled as a *X* ^2^ random variable of *K* degrees of freedom and the likelihood function for the matching perturbed by noise case becomes the *X* ^2^ PDF (eq 3)­

$$L\left(D_{\mathrm{rt}}\;|\;M_{\mathrm{rt}}=1,C_{\mathrm{rt}}=0\right)=\frac{1}{2^{K/2}\Gamma \left(\frac{K}{2}\right)}D_{\mathrm{rt}}^{K/2-1}e^{{-D}_{\mathrm{rt}}/2}$$ 3

### Matching Cross-Peaks Perturbed by a CSP (*M*_*rt*_ = 1, *C*_*rt*_ = 1)

Elements *D*_*rt*_ that correspond to matching cross-peaks and arose from CSPs are modeled as IID Fréchet random variables. Unlike the *X* ^2^ random variable that has a parametrization known *a priori*, the optimal parametrization for this distribution needs to be learned. The likelihood function for this case is by eq 4

4 $$L\left(D_{\mathrm{rt}}\;|\;M_{\mathrm{rt}}=1,C_{\mathrm{rt}}=1;\theta \right)=\frac{\alpha }{s}{\left(\frac{D_{\mathrm{rt}}}{s}\right)}^{-1-\alpha }{e^{-}}^{{\left(\frac{D_{\mathrm{rt}}}{s}\right)}^{-\alpha }}$$

Where α and *s* are the strictly positive shape and scale parameters for the distribution (collectively referred to as the parameter vector θ). On a log–log scale as shown in Figure 1 the description becomes eq 5

5 $$\begin{array}{l} \log L\left(x\;|\;M_{\mathrm{rt}}=1,C_{\mathrm{rt}}=1;\theta \right)=\log\alpha -\left(1+\alpha \right)x-{\left(\frac{s}{e^{x}}\right)}^{\alpha }\\ x=\log D_{\mathrm{rt}} \end{array}$$

At intermediate *D*_*rt*_ the third term dominates leading to the initial sharp upward rise in log probability. At larger *D*_*rt*_ the relationship becomes nearly linear with a slope of −(1 + α)

### Distances between Nonmatching Cross-Peaks (*M*_*rt*_ = 0)

In addition to the two distributions that model elements *D*_*rt*_ between matching cross-peaks, we introduce a third function that defines the likelihood for the elements *D*_*rt*_ between cross-peaks that *do not* match. The form of this likelihood is taken as eq 6

6 $$L\left(D_{\mathrm{rt}}\;\right|\;M_{\mathrm{rt}}=0;\omega )=\frac{1}{\omega }$$

This is a pseudolikelihood based on the observation that the distribution of normalized square distances between nonmatching peaks is approximately uniform at moderate distances from the peak (see Figure 4 below) i.e., the function is constant with respect to *D*_*rt*_. The parameter ω is calculated from a user-supplied input based on the furthest distance the user would expect a peak to move before considering it equally likely that the original peak disappeared and a different peak appeared. It functions as a threshold that guarantees the likelihood of a pair of peaks not matching will exceed the likelihood of the peaks matching at a specified distance. (See the discussion below on Expectation-Maximization for more detail)

Using the three above components we can express the total likelihood as a hierarchical mixture model of the three categories as summarized by eq 7

7 $$L\left(D_{\mathrm{rt}}\;|\;M_{\mathrm{rt}},\theta ,\phi ,\omega \right)=\{\begin{array}{l} L\left(D_{\mathrm{rt}}\;|\;M_{\mathrm{rt}}=1;\theta ,\phi \right),M_{\mathrm{rt}}=1\\ L\left(D_{\mathrm{rt}}\;|\;M_{\mathrm{rt}}=0;\omega \right),M_{\mathrm{rt}}=0 \end{array}$$

Where *L*(*D*_*rt*_ | *M*_*rt*_ = 1;θ, ϕ), the matching likelihood, is the mixture of the *X* ^2^ and Fréchet distributions, which describes the pdf of *D*_*rt*_ given that the corresponding cross-peaks match and is defined as in eq 8

8 $$L\left(D_{\mathrm{rt}}\;|\;M_{\mathrm{rt}}=1;\theta ,\phi \right)=L\left(D_{\mathrm{rt}}\;|\;M_{\mathrm{rt}}=1,C_{\mathrm{rt}}=1;\theta \right)\phi +L\left(D_{\mathrm{rt}}\;|\;M_{\mathrm{rt}}=1,C_{\mathrm{rt}}=0\right)\left(1-\phi \right)$$

Where ϕ, bounded on [0,1], describes the relative contribution of the two components to the matching likelihood. Together this provides a model allowing the evaluation of the likelihood *L*(*D*_*rt*_ | *M*_*i*,*rt*_, θ, ϕ, ω) for any given set of *M*_*i*_,θ,ϕ,ω. Assuming that the likelihood *L*(*D*_*rt*_ | *M*_*rt*_, θ, ϕ, ω) is independent among all *D*_*rt*_ then the likelihood of the entire matrix *D*, *L*(*D* | *M*_*i*_, θ, ϕ, ω) can be calculated as the product of all *L*(*D*_*rt*_ | *M*_*i*,*rt*_, θ, ϕ, ω) for every combination of *r* and *t*.

### Sampling the Posterior Distribution *P*(*M* | *D*,θ,ϕ,ω)

With the likelihood model outlined above, we employed a modified version of the sequential Monte Carlo (SMC) approach outlined in.^12^ The sampling algorithm is summarized here with the details outlined in the Supporting Information. In brief, the SMC algorithm decomposes the sampling process as an autoregressive set of decisions with a single decision made for each reference peak. Each reference peak will have the choice to match to any single target peak (from a prefiltered list of candidate target peaks) or to match nothing. To avoid greedy behavior that leads to poor sampling, the probability of each choice depends on their calculated likelihood as well as the likelihoods of the choices made for subsequent reference peaks. This is implemented as a beam search^13^ done on an optimized reference peak decision order. The probability of the drawn sample calculated by the sampler is evaluated against the likelihood by calculating the effective sample size (ESS). At predetermined decisions the ESS is evaluated. If the ESS is too low, the beam search was insufficient to overcome greedy behavior. This triggers an increase in the maximum number of beams and the algorithm backtracks to improve sampling at the poorly sampled reference peaks. If the ESS is sufficient, the sample undergoes stratified resampling.^14^ This process continues until a decision has been made for each reference peak.

### Determining θ, ϕ, and ω via Expectation-Maximization

Though we have developed a method for sampling the posterior distribution of *M*, this distribution and its SMC sampler is parametrized by θ, ϕ, and ω, the true values of which are not known *a priori*. It is possible to treat these parameters in a fully Bayesian fashion and determine their posterior distributions; however, as these quantities are only of indirect interest, it is sufficient to determine the maximum likelihood estimates (MLE) and the maximum *a posteriori* (MAP) estimate for θ, ω and the mixture model weight ϕ, respectively. In expectation-maximization (EM) optimization, the posterior distribution of matching matrices is sampled, and the sampled matching matrices are then used as inputs to the target likelihood function to optimize the parameters. The algorithm begins with initial estimates for θ, ϕ, and ω. The initial estimate of θ is chosen by collecting the smallest value *D*_*rt*_ at each value of *r*, eliminating all values less than three and finding the MLE for the Fréchet distribution parameters with respect to these elements, bounded such that the shape parameter is greater than or equal to 1. For the mixture weight parameter ϕ which will ultimately be estimated via MAP, we define a prior distribution using a beta distribution and use its mean as the initial estimate for the MAP value. The parameters of the beta distribution (α, β) are influenced by the expected characteristics of the matching problem. The user provides a rough estimate of what fraction of reference cross-peaks are expected to be perturbed by CSPs. The parameters of the β distribution are calculated such that its mean will be equal to that value. The standard deviation of the distribution is set (by default) to two times that of the distribution mean but can be scaled with a user-supplied variance scaling factor. If either α or β are calculated such that it is less than one, both parameters are rescaled such that the mean of the beta distribution is maintained, but both α and β are greater than or equal to one which is necessary for a proper MAP update during EM. For ω, the user supplies an estimate for the largest CSP expected. Using θ and ϕ, the matching likelihood at the normalized distance squared corresponding to the largest CSP is calculated. ω is calculated such that the matching and the nonmatching likelihoods are equal at the largest expected CSP.

### Expectation Step

With initial estimates for θ, ϕ, and ω, the sampling strategy described above is used to generate a set of matching matrices, M . The posterior probability of matching between any two cross-peaks *r* and *t* can be estimated using M by calculating the frequency at which a match between each reference and target peak occurs in the sample according to eq 9

9 $$P\left(M_{\mathrm{rt}}=1\right)\approx \frac{1}{J}\sum_{j=0}^{J}M_{j,\mathrm{rt}}$$

Where $M_{j,\mathrm{rt}}$ refers to the element at row *r* and column *t* for, the *j*th matching matrix in sample M of size *J*. In addition, we can calculate the posterior probability describing that any *D*_*rt*_ arose from a CSP given that reference peak *r* and target peak *t* match. This is calculated via eq 10

10 $${P(C}_{\mathrm{rt}}=1\;|\;M_{\mathrm{rt}}=1)=\frac{\phi L\left(D_{\mathrm{rt}}\;|\;M_{\mathrm{rt}}=1,C_{\mathrm{rt}}=1;\theta \right)}{L\left(D_{\mathrm{rt}}|M_{\mathrm{rt}}=1;\theta ,\phi \right)}$$

### Maximization Step

With the posterior sample of matching matrices and the posterior CSP probabilities, MAP values for ϕ, and ω can be updated. In effect, ϕ is the parameter of a categorical distribution (with a range over two categories). Since the β distribution prior is conjugate with respect to the posterior distribution of ϕ, it can thus be used within a closed form expression to provide a regularized update according to eq 11

11 $$\phi^{\mathrm{new}}=\frac{\beta +\sum_{r=1}^{R}\sum_{t=1}^{T}{P(C}_{\mathrm{rt}}=1\;|\;M_{\mathrm{rt}}=1)P\left(M_{\mathrm{rt}}=1\right)-1}{\alpha +\beta +\sum_{r=1}^{R}\sum_{t=1}^{T}P\left(M_{\mathrm{rt}}=1\right)-2}$$

Where α, β are the parameters of the prior distribution of ϕ.

In the case of θ there is no closed form expression allowing for a direct calculation of an updated MLE. Instead, we approximate the solution numerically by finding θ with the maximum likelihood under the posterior distribution of matching matrices. This can be done by optimizing θ such that the product of the sampled matching matrix likelihoods is maximized as summarized by eq 12

12 $$\theta^{\mathrm{new}}=\underset{\theta }{\arg\max}\prod_{j=1}^{J}L\left(D\;\right|\;M_{j},\theta ,\phi ,\omega )$$

with the goal of finding θ with the maximum likelihood with respect to the sample. After θ and ϕ are updated, ω is updated to the value of ω that makes the matching likelihood and nonmatching likelihoods equivalent at the maximum expected CSP.

With these updated parameters (θ, ϕ, and ω), a new round of sampling the posterior distribution can commence, followed by an additional round of parameter updates, and continuing until parameter convergence. Once the converged parameters have been identified, the posterior distribution of matching matrices can be sampled and posterior probabilities of individual matches calculated. A flowchart of the EM algorithm is outlined in SI Figure 1.

## Materials and Methods

### NMR Spectroscopy

Uniformly ^15^N- ^13^C- and ^15^N labeled human IL-1β was expressed recombinantly in *E. coli* BL21­(DE3) cells and purified essentially as described elsewhere.^15^ IL-1β was encapsulated in LDAO/10MAG reverse micelles^16^ in hexane with 2% D_14_—hexane at an aqueous concentration of 5 mM and a water loading of 15 (corresponding to a concentration relative to the sample volume of 0.1 mM) as described previously.^15^ NMR spectra were acquired at 25 °C on an 800 MHz four channel Bruker NEO Avance NMR spectrometer equipped with a cryogenically cooled probe. Three-dimensional BEST-TROSY^17^ HNCO experiments were collected with 512 × 50 × 22 complex points and 48 scans per free induction decay and two-dimensional ^15^N-TROSY HSQC experiments were collected with 1024 × 64 complex points and 16 scans per free induction decay on ^15^N labeled protein. Data were processed in NMRpipe^18^ and analyzed with POKY.^19^ ^15^N-TROSY HSQC spectra were processed with the same apodization as the ^1^H and ^15^N dimensions of the 3D HNCO. Automated peak picking was conducted using UniDecNMR.^2^ Peak centers were subsequently refined using the *voigt_fit* software in the DEEP Picker^1^ software suite. The resulting peak lists were then used directly without further curation for peak matching.

U–^2^H,^15^N,^13^C [MLV–^13^CH_3_] labeled His-tagged scFv of the fluorescein antibody 4–4–20 was expressed in *E. coli* BL21­(DE3) cells during growth on minimal media containing 99% D_2_O supplemented with 2 g/L of deuterated glucose [^13^C–D7] and 1 g/L of ^15^NH_4_Cl (Cambridge Isotopes), isolated from inclusion bodies, and purified by affinity chromatography on a HisTrap (Cytiva) nickel column. The protein was refolded by dropwise dilution. Details of the expression, purification and refolding will be presented elsewhere. The scFv:fluorescein complex was 0.2 mM in protein prepared in 10 mM sodium phosphate, 50 mM NaCl at pH 6.0 with 5% (v/v) D_2_O, 0.02% (w/v) NaN_3_.

Triple resonance experiments were carried out at 25 °C on an 800 MHz (^1^H) four channel Bruker NEO Avance NMR spectrometer equipped with a cryogenic probe. Chemical shift resonances were referenced with sodium 2,2-dimethyl-2-silapentane-5-sulfonate (DSS). Indirect dimension was acquired using NUS with Poisson gap distribution sampling (10% of Cartesian grid) and spectra were reconstructed using hmsIST^20^ and processed with NMRpipe in NMRBox.^21^ Resonance assignments for antifluorescein scFv 4–4–20 and hParkin­(ΔUbl) were obtained using BARASA^22^ and will be reported elsewhere.

A gene coding for human Parkin lacking the ubiquitin-like (Ubl) domain and associated linker, hereafter termed hParkin­(ΔUbl), corresponding to (SS) followed by residues 135 through 465 of the canonical isoform (Swiss-Prot O60260–1) was prepared in a His-SUMO tag with TEV cleavage site in a pET32b vector as described previously^23^ and expressed during growth on minimal media prepared with ^15^NH_4_Cl and D_2_O. The protein was refolded and purified as described previously.^23^ NMR spectroscopy was performed on Parkin at a concentration of 0.160 mM in 50 mM Tris pH 7.8, 100 mM NaCl, 5 mM TCEP, 2.5% glycerol, 10% D_2_O. The sample bound *in trans* to its native UBL domain was mixed with UBL domain to a final concentration of 0.18 mM. ^15^N-TROSY HSQC spectra were acquired at 25 °C on an 800 MHz four channel Bruker NEO Avance NMR spectrometer equipped with a cryogenically cooled probe using 1024 × 128 complex points and 32 scans per free induction decay.

### pHarmony Parameters

For all test cases, peak errors were set to 0.006, 0.015, and 0.0015 ppm for ^13^C, ^15^N and ^1^H dimensions, respectively. For analysis of the spectra derived from the fragment-based ligand discovery screen of IL-1β, the expected maximum CSP was set to 0.15 ppm for 2D matching runs and 0.2 ppm for 3D matching runs. The expected fraction of reference cross-peaks undergoing a CSP was set to 0.1 with a variance scaling factor of 2. For analysis of the triple resonance spectra of the fluorescein scFV, all of which were acquired on the same sample, the expected maximum CSP was set to 0.1 and the expected fraction of CSPs was set to 0.001 with a variance scaling factor of 2. CSPs were calculated by scaling the chemical shifts in the carbon and nitrogen dimensions by a factor of 0.252 and 0.101 respectively before computing the Euclidean distance between cross-peaks.

## Results

### Harmonizing Cross-Peak Sets Anticipated to Be Closely Identical

Perhaps the simplest and most efficient application of the pHarmony algorithm would be the rapid comparison of pairs of spectra containing common dimensional elements. This might include triple resonance spectra such as a ^15^N TROSY and HN­(CO)­CA where the spectra possess a set of axes reporting on identical resonances. Though relatively simple to manually interpret, a rapid analysis that provides a statement of confidence for the matching of cross-peaks would significantly decrease the burden of manual analysis by highlighting those mappings of concern.

To evaluate this type of mapping, we employ a 30 kDa scFV construct of the fluorescein antibody 4–4–20,^24^ which has been assigned using BARASA.^22^ The assignments will be reported in detail elsewhere. This construct of 255 amino acids gives high quality triple resonance spectra as evidenced by the ^15^N-TROSY HSQC spectrum (Figure 2). Cross-peaks in the two-dimensional ^15^N-TROSY HSQC and three-dimensional TROSY triple resonance spectra (Table 1) were manually picked. The performance of pHarmony on this data set was evaluated using two criteria: accuracy (precision) and completeness (recall).

**Figure 2:**
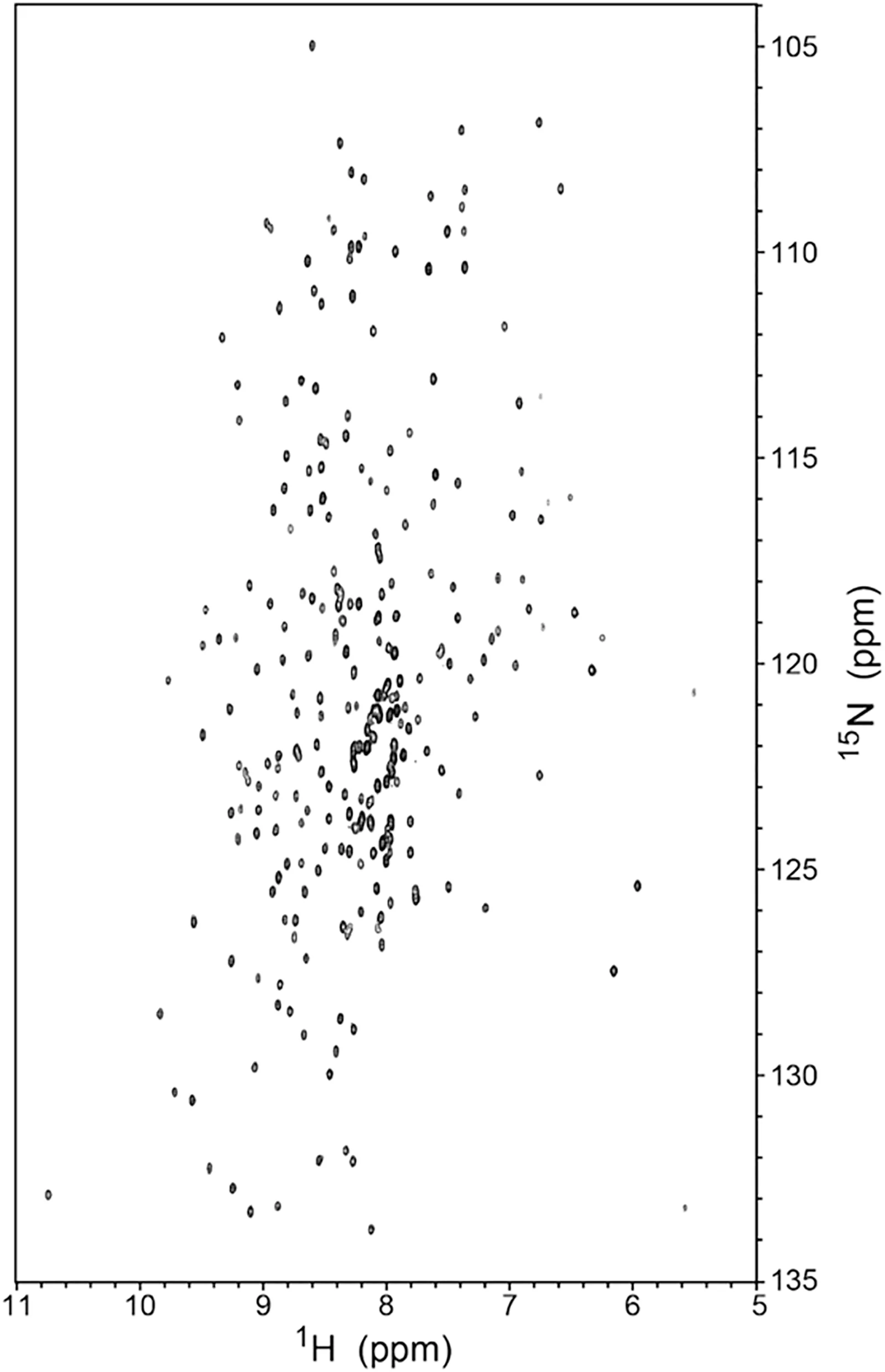
^15^N-TROSY HSQC spectrum of the scFv construct of the antifluorescein antibody 4–4–20 obtained at 800 MHz (^1^H) and 308 K.

**Table 1:** Performance of Tested Algorithms on Different Data Sets^a^

Algorithm	scFV 4–4–20^b^ all spectra	IL-1β–3D HNCO	IL-1β–2D HNCO	IL-1β–2D TROSY HSQC	IL-1β–TROSY HSQC Auto^c^	hParkin(ΔUbl) TROSY HSQC
pHarmony^d^ ^,^ ^e^	100/98.5	99.9/98.5	99.3/92.2	98.6/93.6	97.6/93.3	98.9/76.4
NN^e^	99.8/99.7	99.7/99.1	97.7/94.2	97.3/95.7	95.3/96.1	95.7/82.5
PICASSO	94.0/96.3	N/A	94.0/95.0	93.4/95.8	85.8/91.6	87.6/87.9

a Performance is indicated as percent accuracy/completeness.

b Mean values across all cross-peak sets defined in Figure 3

c Using automatically picked cross-peak sets, see Materials and Methods section.

d Matches made with a posterior probability of >0.95 were accepted as matched by pHarmony.

e The maximum expected CSP for pHarmony and NN were set to 0.15 ppm for 2D experiments and 0.20 ppm for 3D experiments (proton units).

Accuracy is defined as the ratio of “true” matches made by the algorithm to total matches made by the algorithm. Completeness is defined as the ratio of correct matches made by the algorithm to the total number of “true” matches. True matches were determined by manual annotation (where the triple resonance experiments provided unequivocal identification). Furthermore, since each match made is assigned a posterior probability, accuracy and completeness of matches can be evaluated as a function of match posterior probability, which serves as a confidence metric.

The performance of pHarmony when matching triple resonance spectral pairs of the scFv construct of the antifluorescein antibody 4–4–20 is shown in Figure 3. A match was determined for each reference peak by selecting the highest probability matching target peak. These matches were then binned as a function of posterior probability. Employing a 95% posterior probability cutoff, the accuracy was 100% for the matches made in all cases. Completeness was in the range of 96–98%, meaning that the algorithm was missing very few of the possible matches at very high accuracy. The accuracy as a function of posterior probability did not appreciably follow the line of identity. However, this is likely due to high variance in the sampled accuracy over the low probability bins as the number of matches in posterior probability bins below 0.95 were all less than 12 per bin.

**Figure 3:**
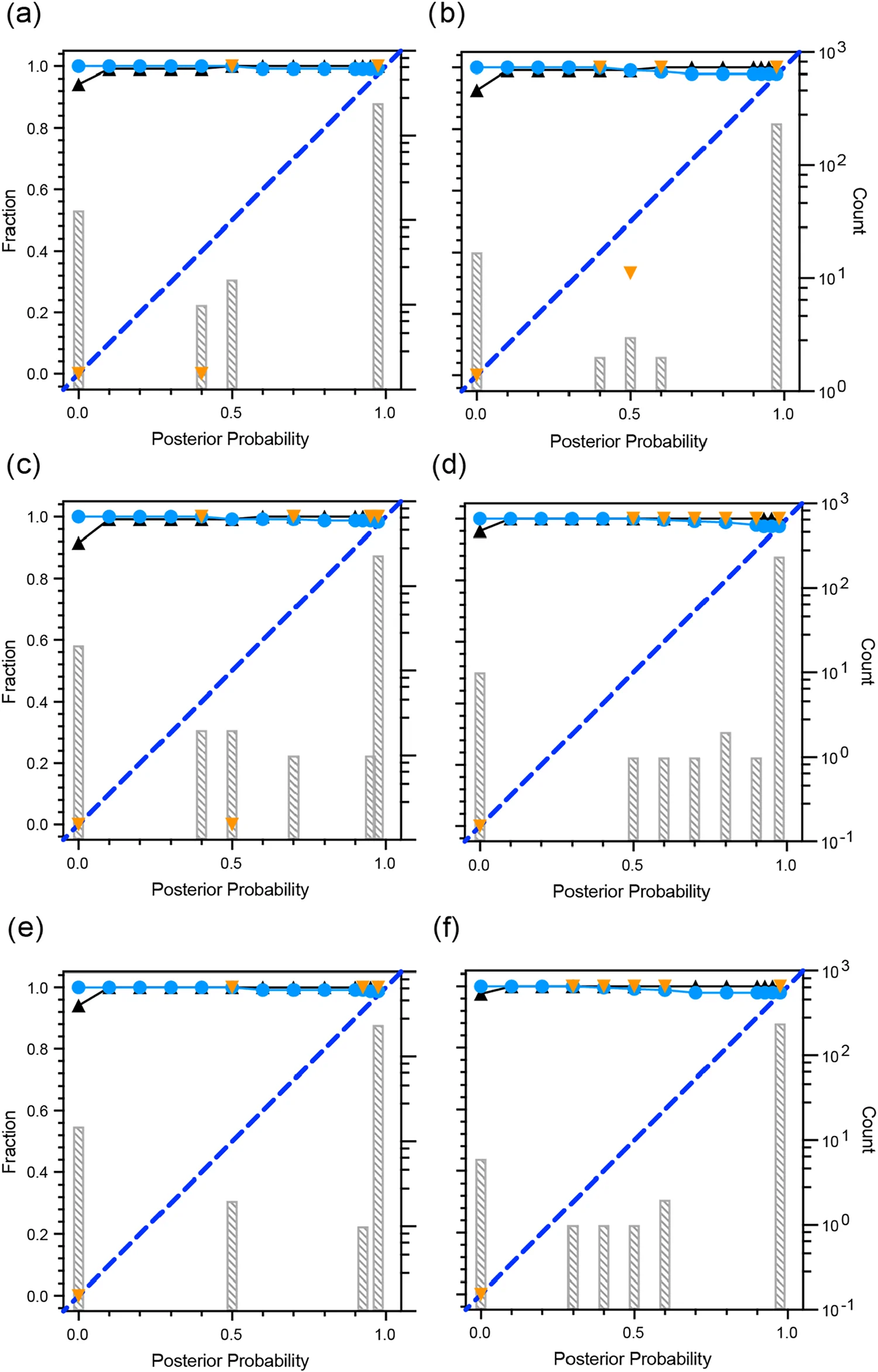
Performance of pHarmony in cross-peak matching between triple resonance spectra. Manually peak picked spectra of the 30 kDa antifluorescein scFv 4–4–20. Spectra were matched using only the chemical shift information from the ^15^N and ^1^H chemical shift coordinates. Shown for a given posterior probability are the accuracy of matches (orange triangles), cumulative completeness (blue circles) and cumulative accuracy (black triangles). Cumulative accuracy is defined as the accuracy of all matches at the indicated and all higher posterior probabilities. The accuracy data series omits points for empty posterior probability bins (i.e., no matches were made at that posterior probability range). Cross-peaks of the ^15^N-TROSY HSQC spectrum (reference) matched with ^1^H–^15^N dimensions of cross-peaks in the target TROSY HNCO (Panel a), TROSY HN­(CO)­CA (Panel b), TROSY HN­(CO)­CB (Panel c). The TROSY HNCO spectrum (reference) was matched with the target TROSY HN­(CO)­CA (Panel d) and TROSY HN­(CO)­CB (Panel e) spectra. The TROSY HN­(CO)­CA (reference) was matched with the target TROSY HN­(CO)­CB spectrum (Panel f). Spectra were matched using only the chemical shift information from the ^15^N and ^1^H chemical shift coordinates. The vertical dotted lines indicate a proposed confidence cutoff (0.95) for accepting matches.

### Harmonizing Cross-Peak Sets Anticipated to Be Significantly Different

A more subtle and difficult task is the mapping of cross-peak sets that contain large numbers of chemical shift differences. This situation can arise in numerous instances such as pH or other solvent changes, ligand titrations, temperature and pressure changes. To test pHarmony in this context, we utilized results from a fragment-based ligand discovery campaign of interleukin-1β (IL-1β) followed by multidimensional NMR spectra. This screen employed the reverse micelle encapsulation strategy to enhance detection of relatively weak binding fragments.^15^ Details of the screen will be presented elsewhere. A rule-of-three (Ro3) fragment library customized for screening in the context of proteins encapsulated within reverse micelles (RM) was employed. ^15^N-TROSY HSQC spectra of hParkin­(ΔUbl) (333 residues, 37 kDa) in its free state and complexed *in trans* with its native UBL domain were also used to provide a test containing broader peaks with overlap. Details of the NMR study of Parkin and its associated domains will be reported elsewhere.

TROSY-HNCO and ^15^N-TROSY HSQC spectra were acquired on 11 samples of IL-1β encapsulated in 10MAG/LDAO reverse micelles. Each sample of IL-1β contained 5 different fragment compounds each present at an effective aqueous concentration of 40 mM. Two samples of IL-1β contained no fragments as a control. Each cross-peak in all spectra were manually annotated to serve as the ground truth. The carbon dimension of the HNCO was used to unambiguously track cross-peaks that moved in regions of degeneracy within the ^15^N-correlation (sub)­spectrum. The algorithm was tested in four ways. The first three used manually picked peak lists and used to test (1) the ability to match cross-peaks between two HNCOs using all three dimensions (3D-TROSY HNCO mode); (2) the ability to match cross-peaks between 3D HNCOs using only the amide dimensions (2D-TROSY HNCO mode); and (3) the ability to match cross-peaks between two HSQCs (2D-^15^N-TROSY mode). The fourth test relied on automatically picked cross-peaks on HSQCs (2D-^15^N-TROSY -Auto mode). In each mode, the algorithm was tested for its ability to match cross-peaks between all possible pairs of spectra (excluding matching a cross-peak list with itself). Additionally, since the algorithm treats reference and target cross-peaks differently, each pair of cross-peak lists was evaluated with each list as the target and the reference, leading to 110 runs for each mode. To evaluate peak matching in the context of a larger protein system, spectra of the hParkin­(ΔUbl) with and without its native UBL domain added *in trans* was also collected. UBL was added at a concentration sufficient to saturate hParkin­(ΔUbl). The matching was done with the apo sample as the reference and the UBL-containing sample as the target and vice versa.

Matching two IL-1β 3D-TROSY HNCO spectra offers the opportunity to illustrate the distributions of normalized square distances of a more complex, real-world example than that shown in Figure 1. Figure 4 shows the three distribution classes, effectively noise and noise plus CSP matched distributions and the assumed unmatched function for comparison to two three-dimensional HNCO spectra of encapsulated IL-1β in the presence of two different mixtures of fragments.

**Figure 4:**
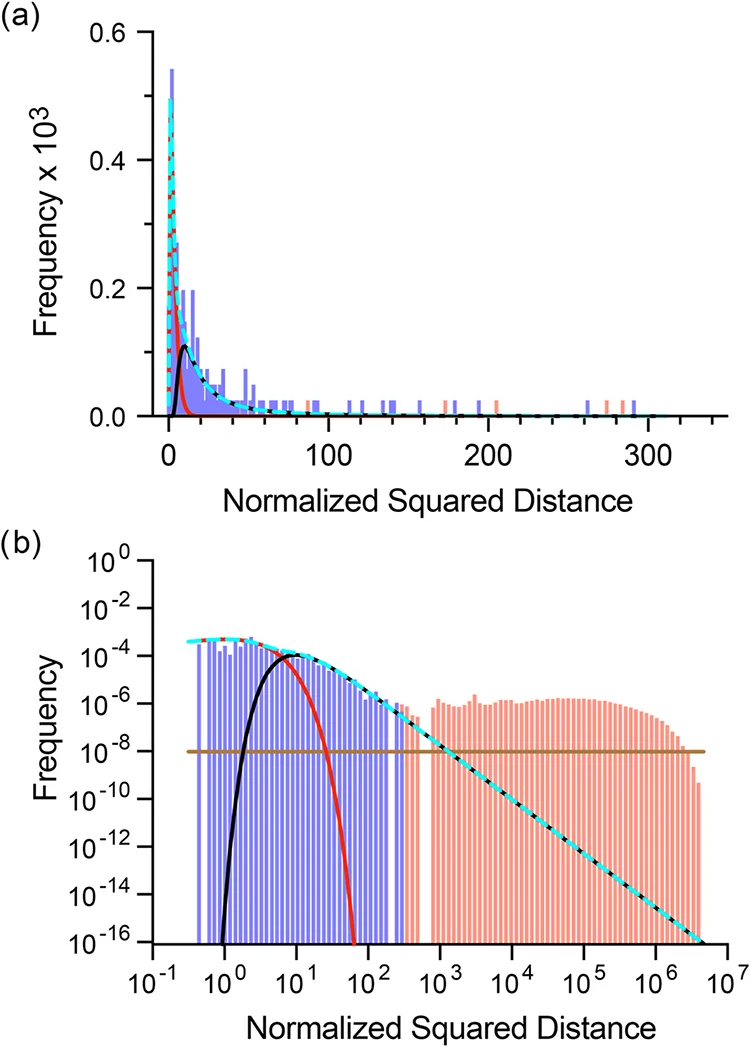
Example of distributions of normalized square distances of three-dimensional HNCO spectra. Pairwise normalized square distances between two IL1β 3D HNCO spectra were binned, with each distance classified as being either between peaks that match or do not match by the algorithm (at EM convergence). Blue-violet bars correspond to distances between matched peaks and red bars correspond to distances between peaks that were not matched. The solid red line corresponds to the component of the likelihood function for distances between matched peaks pair that did not undergo a CSP (Chi squared distribution). The solid black line corresponds to the component of the likelihood function for distances between matched peak pairs that undergo a CSP (Frécheet distribution). The dashed cyan line corresponds to the matched probability distribution. The solid brown line corresponds to the pseudolikelihood function for distances between pairs of peaks that do not match. In this example, there are 198 reference peaks being compared to 207 target peaks giving 40,986 normalized squared distances. The top panel (a) is plotted in linear space showing the region containing all distances between matching pairs. The bottom panel (b) is plotted in log–log space showing all pairwise distances.

The accuracy and completeness of pHarmony as a function of match posterior probability for each of the testing modes is summarized in Figure 5. Among the testing modes involving manually picked cross-peaks, the 3D-TROSY HNCO (Figure 5a) gave the best results both in terms of accuracy and completeness when considering the high posterior probability matches. The 2D- TROSY HNCO and HSQC testing modes (Figure 5b and c, respectively) saw a slight decrease in the accuracy and completeness among the high posterior probability matches. However, if accepting matches with an estimated posterior probability of 0.95 or greater, matches between cross-peaks of manually picked spectra have an accuracy of at least 98% and represent 93% of the “true” matchings. Overall performance using automatically picked cross-peaks on 2D HSQCs was similar to performance on the manually picked peak lists, with 97% of high probability matches being correct and 93% of all possible matches being marked as high confidence.

**Figure 5:**
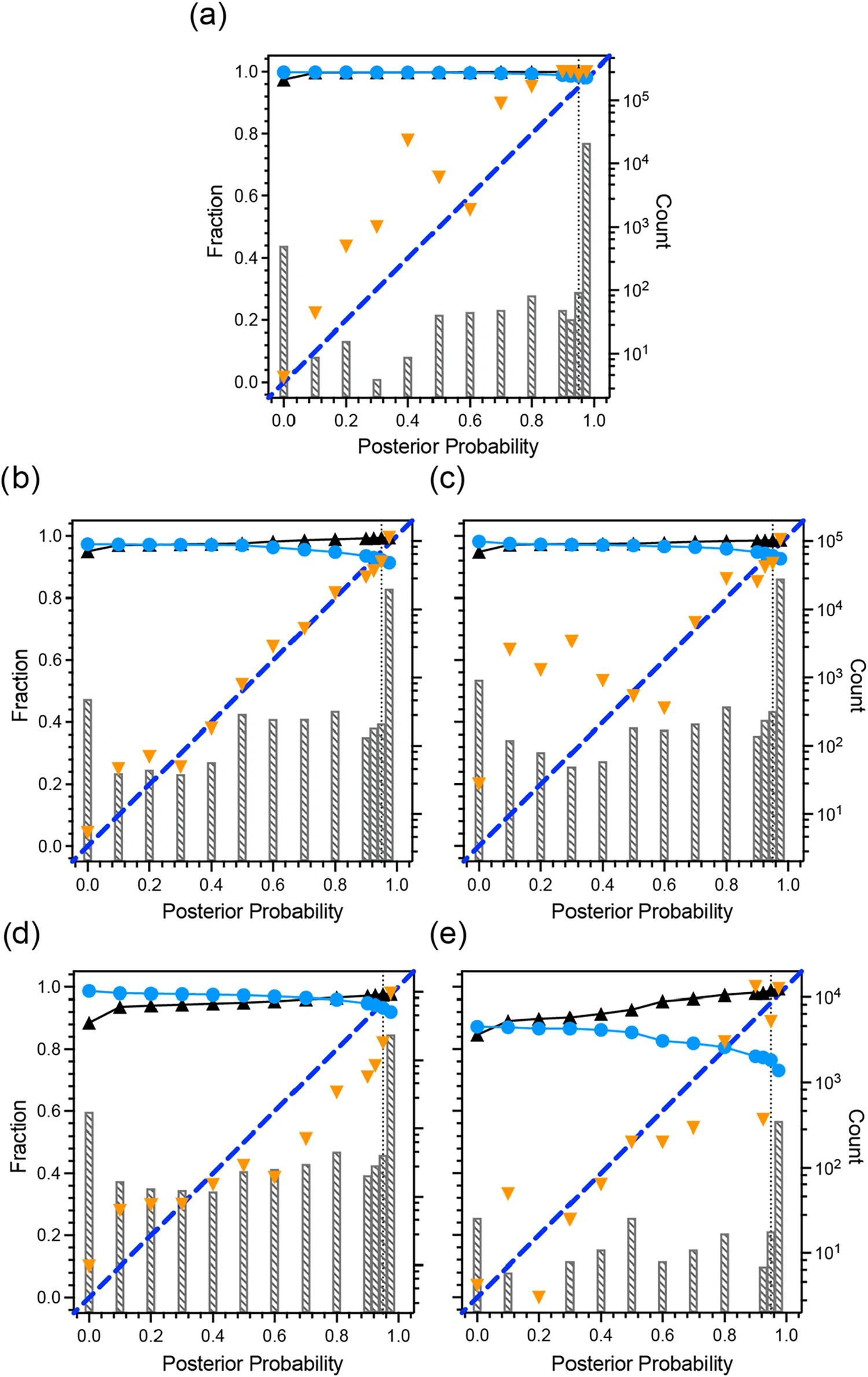
Accuracy and completeness of pHarmony as a function of matching posterior probability across five different testing modes. Matches are binned by posterior probability. Matches of manually peak picked IL-1β three-dimensional HNCO cross-peaks (Panel a), manually picked IL-1β two-dimensional first ^13^C-increment of the HNCO cross-peaks (Panel b), manually picked IL-1β two-dimensional ^15^N-TROSY cross-peaks (Panel c), automatically picked IL-1β two-dimensional ^15^N-TROSY HSQC cross-peaks (Panel d) and manually picked hParkin­(ΔUbl) two-dimensional ^15^N-TROSY HSQC cross-peaks (Panel e). Shown for a given posterior probability are the accuracy of matches (orange triangles), cumulative completeness (blue circles) and cumulative accuracy (black triangles). Hatched bars show the number of matches placed in each bin. The vertical dotted lines indicate a proposed confidence cutoff for accepting matches (0.95).

The performance of pHarmony against these CSP containing data sets was compared against two benchmarks, the peak matching algorithm a simple nearest neighbor algorithm (NN) with a maximum allowed CSP and the existing algorithm PICASSO. NN was run as a comparison since the nature of the pHarmony likelihood function and sampling algorithm heavily prioritizes nearest neighbors when matching. Thus, a comparison against NN would demonstrate possible advantages of pHarmony’s probabilistic approach. The performance of PICASSO, which is designed for 2D 15N HSQC type experiments was evaluated on the manually picked 2D HNCO, 2D HSQC and automatically picked 2D HSQC data sets. Testing was done using the “RA” algorithm and the default nitrogen dimension scaling factor of 5 which are the default parameters for matching with PICASSO when backbone assignments are not known. The algorithm that matches without consideration of backbone assignments was chosen as the peak lists used contained both backbone and side chain amide peaks, as well as a fair number of unassigned peaks that are likely originating from backbone resonances due to slow exchange. The NN algorithm was run on all data sets and used a CSP cutoff that matched the maximum expected CSP parameter passed to pHarmony. A comparison in the performance of pHarmony and other algorithms is outlined in Table 1.

For the 3D data set, performance characteristics between pHarmony and NN were most similar. In the two-dimensional data sets pHarmony maintains consistently higher accuracy rates than NN, corresponding to an approximate halving of the error rate (incorrect matches made between peaks). This comes at the expense of deeming correct matches detected by NN as ambiguous leading to lower completeness. The accuracy of PICASSO was generally lower. On the IL-1β data sets, performance on certain spectral pairs had accuracy of ∼94% for manually picked 2D spectra. The accuracy was below 86% in the case of automatically picked peaks, with the completeness of ∼92%. For the Parkin 2D spectral data set, PICASSO had the highest completeness but the lowest accuracy.

To examine the relative performance of pHarmony, NN and PICASSO in more detail, the accuracy of the algorithms were examined as a function of the magnitude of the CSP (Figure 6). As CSPs grow the possibility of ambiguity increases potentially increasing the odds of mismatched peaks. All algorithms showed a decrease in accuracy as a function of CSP. The accuracy for NN and pHarmony were similar when matching 3D cross-peaks across all CSPs, with slightly higher accuracies at moderate (∼0.05 ppm) CSPs. pHarmony achieved slightly higher accuracy than NN at moderate CSPs. The accuracy of pHarmony was less sensitive to moderate CSPs than NN when matching 2D cross-peaks. However, at large CSPs, NN and pHarmony behaved similarly. PICASSO gave a larger decrease in accuracy with increasing CSP than either pHarmony or NN.

**Figure 6:**
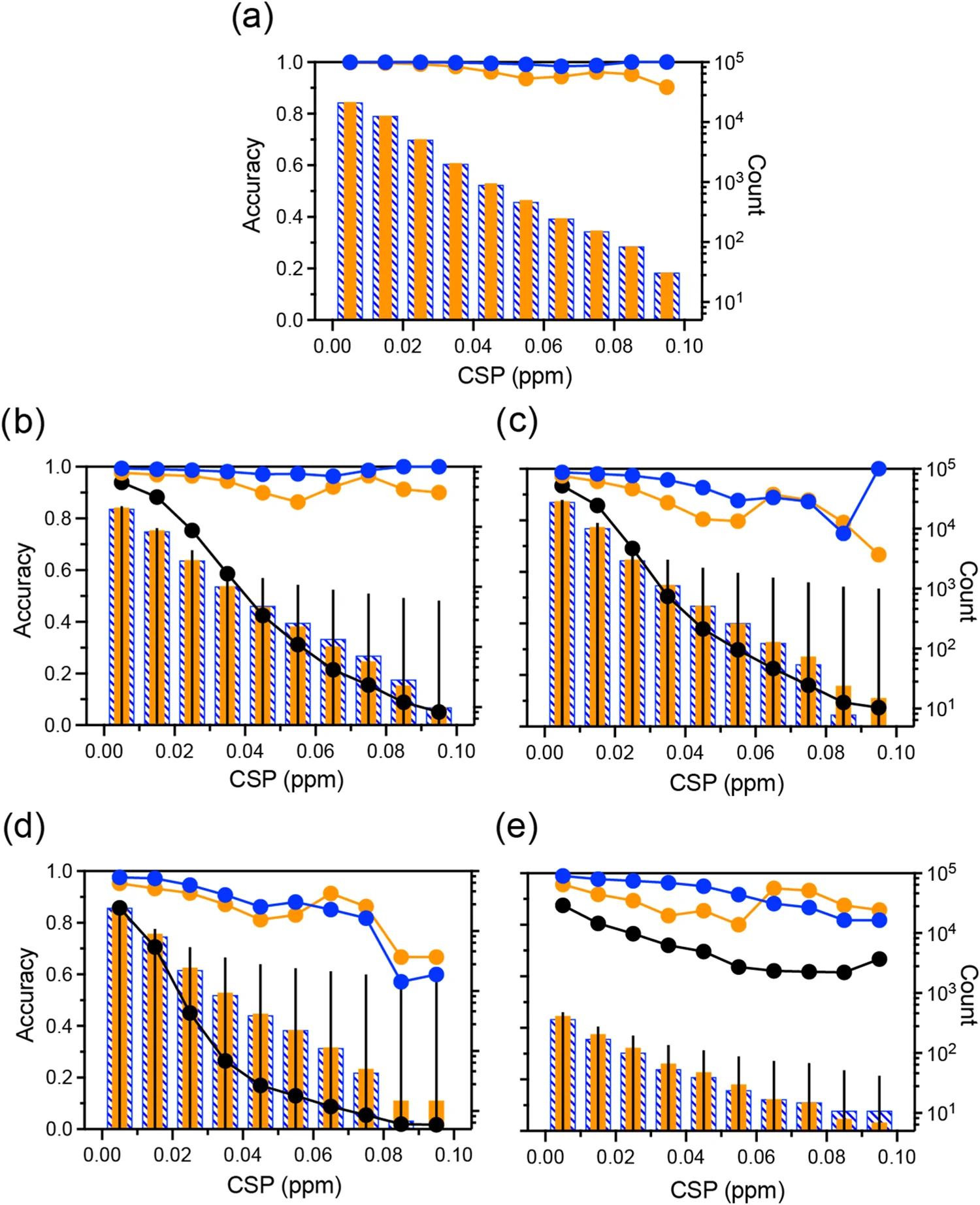
Accuracy as a function of reverse cumulative CSP (each bin contains all matches made by the respective algorithm with CSPs of the indicated magnitude or larger). Panel a—matches between 3D TROSY-HNCO IL-1β spectra using all three dimensions. Panel b—matches made between 3D TROSY-HNCO IL-1β spectra using only the amide dimensions. Panel c—matches made between 2D ^15^N TROSY IL-1β spectra. Panel d—matches made between 2D ^15^N TROSY IL-1β spectra with automatically picked peaks. Panel e—matches made between 2D ^15^N TROSY hParkin­(ΔUbl) spectra. Accuracy for matches made by pHarmony, NN and PICASSO are shown in blue, orange and black circles, respectively. The count of matches made by each algorithm are shown by the blue hatched bars, orange solid bars, and black solid bars for pHarmony, NN, and PICASSO, respectively.

In addition to accuracy, there was a concern that the pHarmony algorithm could have difficulty in confidently detecting larger CSPs. Though large CSPs tend to be less common in most contexts, cross-peaks that undergo large CSPs are often of the greatest interest. By the nature of the probability model, the likelihood of two cross-peaks matching decreases as the distance between them increases. At sufficiently large CSPs, pHarmony may begin to spread probability density across several possible matches of similar distance as well as the no match condition. Figure 7 shows the completeness of matches made by pHarmony, NN and PICASSO. Matching 3D cross-peaks had the best results in terms of detecting long-range CSPs. Understandably, both pHarmony and NN are less capable when evaluating 2D cross-peak sets. pHarmony shows generally higher completeness in the 2D TROSY HNCO data sets than NN. Both show similar completeness in each 2D ^15^N TROSY data set. PICASSO showed the highest completeness at nearly all CSPs but was accompanied by a significant loss of accuracy with increasing CSP (i.e., generation of false matches).

**Figure 7:**
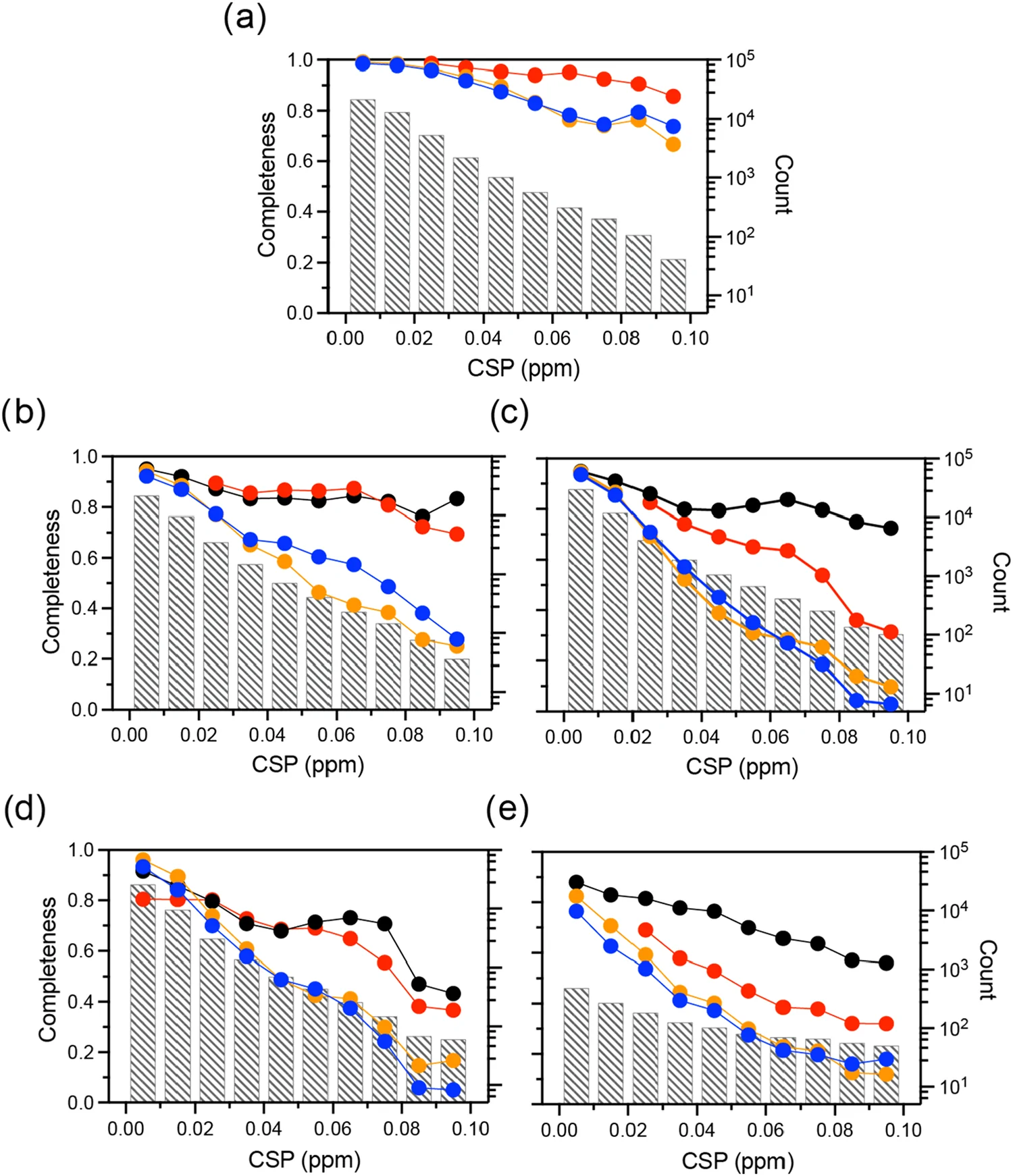
Completeness as a function of reverse cumulative CSP (each bin contains all true matches with CSPs of the indicated magnitude or larger). Panel a—matches between 3D TROSY-HNCO IL-1β spectra using all three dimensions. Panel b—matches made between 3D TROSY-HNCO IL-1β spectra using only the amide dimensions. Panel c—matches made between 2D ^15^N TROSY IL-1β spectra. Panel d—matches made between 2D ^15^N TROSY IL-1β spectra with automatically picked peaks. Panel e—matches made between hParkin­(ΔUbl) 2D ^15^N TROSY spectra. Completeness for matches made by pHarmony, NN, and PICASSO are shown in blue, orange, and black, respectively. Completeness of pHarmony detection of significant reference peak movement (defined as 0.025 ppm or greater) via marginal probability integration is shown in red. A match was considered made by pHarmony if it possessed a posterior probability of 0.95 or greater. A CSP was considered detected by pHarmony if the sum of the marginal matching probabilities between a reference peak and the set of target peaks that would be considered significantly perturbed (CSP > 0.025) was 0.95 or greater. The count of true matches in each bin is shown by the gray hashed bars.

In addition to the examination of direct matches, knowledge of exact matches between peaks may only be of indirect interest in certain contexts. In the context of a ligand screening campaign, the goal of peak matching is to identify which reference peaks underwent a significant CSP, not to necessarily determine exactly where each peak went. It is in this context that a probabilistic approach has considerable value. It is not necessary that a hit detection algorithm confidently identify a single, definite match for a reference peak in a target spectrum to determine whether a CSP has occurred. Rather, if the algorithm could confidently identify that a reference peak matches any from a subset of target cross-peaks, all of which correspond to a CSP sufficiently large to be significant, then the user could confidently assert that a peak was significantly perturbed even if there is some uncertainty exactly as to how it was perturbed. Answering this question for a given reference spectrum peak requires its marginal matching probability distribution over the possible target cross-peaks. As the algorithm samples the *joint* matching probability distribution, estimation of the marginal distribution involves integrating over the sampled matching matrices to determine the frequency with which the reference peak in question matched each target peak. If a large percentage (>95%) of the marginal probability mass corresponded to target peak matches above a CSP significance cutoff (>0.025 ppm), then the user could safely conclude that the peak was perturbed above the CSP significance cutoff without exactly knowing which target peak it matched. Figure 7 shows when testing the ability of pHarmony to identify significantly perturbed residues as opposed to finding exact matches, a rise in completeness was observed for all CSP bins. This was observed across each test data set. Accuracy of the detected CSPs (true positive rate) remained at 96% or greater for all data sets tested.

The performance of pHarmony was also evaluated in the context of different priors. pHarmony accepts a maximum expected CSP, an expected fraction of reference peaks that are perturbed (i.e., Fréchet distributed), and a scaling factor for the expected variance in that fraction. The effect of these priors on algorithm performance is summarized in SI Figures 2 and 3. The expected fraction of perturbed peaks and its variance scaling had a modest impact on final accuracy and completeness. The maximum expected CSP had a considerable effect on the ability to detect large CSPs. Increases in this parameter resulted in small reductions in accuracy at high confidence.

## Discussion

The pHarmony algorithm was able to obtain a high degree of accuracy and completeness on matches between both 2D and 3D spectra when employing manually picked cross-peaks. Performance in terms of both accuracy and completeness was better for the 3D spectra likely due to the extra resolution afforded by the third dimension. Furthermore, the posterior probability of the matches was generally close to the frequency at which the resulting match was correct as shown by the tendency of the accuracy to track the line of identity (Figure 5). This demonstrates that the computed posterior probability is a reliable indicator of confidence in the veracity of a proposed match. Though accuracy as a function of posterior probability for the 3D HNCO test case, as well as in the hParkin­(ΔUbl) ^15^N TROSY data set, has quite a bit of scatter, it should be noted that the number of matches in most bins below posterior probability 0.8 was smaller than 50 with many containing fewer than 10. These small sample sizes likely contributed to high variance in the estimated accuracy at low posterior probabilities. These observations support the validity of the model used to compute matching likelihoods, as it demonstrates that the calculated posterior probability closely approximates the quantity that it is trying to model which is the probability a proposed match between a reference peak and target peak is correct. Posterior probability bins from 0.1–0.4 in the IL-1β 2D ^15^N TROSY data set with manually picked peaks have higher than expected accuracy. This is attributable to the presence of merged peaks becoming distinct peaks between certain spectral pairs. Since the algorithm enforces one to one matching, the matching probability of one of the resolved peaks to the merged peak tends to be <0.5, even though it is a correct match. This is evident when comparing against the 2D TROSY-HNCO test results. Since the peaks were resolved in 3D, the positions of the amide resonances are well determined. Thus, the 2D TROSY-HNCO peak lists have near perfect knowledge of all amide peak positions, even in regions of high degeneracy. In this circumstance where all peak positions are known with high accuracy, the estimated posterior probability tracks the accuracy with good agreement. Introduction of ambiguity in peak position and existence (e.g., merging of cross-peaks in the manually picked 2D ^15^N TROSY spectra) leads to deviations from the line of identity and increases as additional peak picking errors are introduced, for example, by automated peak picking.

For circumstances when peak positions were unambiguously identified (e.g., the 3D and 2D TROSY-HNCO IL-1β test sets), there was a modest dependency of accuracy (pHarmony and NN) on the CSP of the match. The ambiguity introduced by the loss of the carbonyl dimension had negligible effect on the pHarmony’s accuracy for high CSP matches, though NN experienced a small drop in accuracy for matches with CSPs of approximately 0.05 ppm. In contrast, the pHarmony responded to the loss of carbonyl resolution by making fewer matches, especially those with higher CSPs leading to lower completeness. NN shows a similar trend. In these 2D TROSY- HNCO test cases, pHarmony generally outperformed NN in terms of accuracy and completeness at moderate to large CSPs. This is likely due to the subglobal nature of pHarmony’s beam search for peak matching decisions allowing the algorithm to detect distant matches. Completeness is further improved when the problem is restricted to significant CSP detection. This implies that pHarmony, under testing conditions of well determined peak positions, is still assigning most of the matching density to target peaks. This suggests that the loss of completeness when making matches is driven mostly by sampling matches to multiple target candidates with similar likelihoods as opposed to being frequently sampling unmatched states. In contrast, PICASSO lacks a statement of confidence and therefore has no knowledge of the existence of solutions of similar likelihood which results in more false matches (see Figures 6 and 7).

The performance of pHarmony changes with the introduction of ambiguous peak positions such as merged peaks in the case of the manually picked 2D ^15^N TROSY spectra and false peaks in the case of automatically picked 2D ^15^N TROSY spectra. Overall accuracy was superior to that of NN, while completeness was lower, suggesting an overall tendency for pHarmony to be conservative with match decisions. While overall accuracy and completeness remain high for the IL-1β ^15^N TROSY test sets, accuracy and completeness as a function of increasing CSP experienced decreases. The decline in accuracy with CSP was modest with the decline in completeness being substantial. The hParkin­(ΔUbl) 2D ^15^N TROSY data set maintained high accuracy despite increased degeneracy and peak overlap but with significantly lower completeness, suggesting that pHarmony handles ambiguity conservatively by scoring uncertain matches appropriately. Overall accuracy as a function of CSP is generally higher for pHarmony vs NN. This was not always the case for the highest CSPs. However, the match count in these bins were small (<20). The computed accuracy in these bins may have large sample to sample variance. In contrast completeness as a function of CSP was similar between NN and pHarmony, with NN showing a slightly larger completeness for the hParkin­(ΔUbl) data set. Completeness of the pHarmony significant CSP detection was higher than the detection of exact matches. However, the improvement was smaller in the 2D ^15^N TROSY data sets as opposed to the HNCO data sets. This was particularly true for the hParkin­(ΔUbl) 2D ^15^N TROSY data set which experienced very modest improvements. This suggests that ambiguity in peak position and existence leads to an increase in the posterior probability of a reference peak being matched to nothing. As for the 2D TROSY HNCO cross-peak sets, PICASSO maintained the highest completeness at all CSPs, but with relatively low accuracy. Accuracy decreased further upon introduction of spurious peaks generated by automatic peak picking. Interestingly, PICASSO’s accuracy at high CSP was substantially better for the hParkin­(ΔUbl) data set, though still lower than either pHarmony or NN.

The large decline in completeness as a function of CSP observed for 2D data is likely due to a few factors. The ground truth matches that define the total number of possible matches were made with access to 3D spectral data that the 2D peak matching tests lack. When the 3D cross-peak set is evaluated, the ambiguities are generally resolved confirming the utility of three-dimensional spectra. Thus, part of the loss of completeness in the analysis of the 2D cross-peak data is partially due to insufficient information. Peak overlap is another factor, evident by a large decrease in large CSP detection when comparing the 2D HNCO and 2D HSQC data sets. When multiple true peaks overlap but are represented by only one picked “composite” peak, the algorithm often identifies several moderate confidence matches to that single peak. Because the posterior probability is divided among these alternatives, no one match may receive sufficient probability mass to be considered high confidence. This uncertainty can also propagate through the one-to-one matching constraint: peaks competing for the composite peak must place probability elsewhere, often on other possible matches, creating secondary competition with neighboring peaks. The result is a more diffuse posterior distribution and a reduced ability to assign confident matches, lowering completeness.

When choosing the set of prior values to initialize pHarmony, consideration should be made as to prior belief of differences between the spectra. For example, if matching peaks between different spectra acquired on the same or nearly identical sample, perturbations beyond noise should be low. Thus, setting a low maximum expected CSP (<0.1) and a low expected fraction CSP (<0.01) with a low variance scale (<2) biases the model to peak match based on these prior beliefs. If collecting data on samples where numerous perturbations are expected, setting the expected fraction CSP between 0.1 and 0.5 is appropriate. Changing the variance scale in these circumstances is likely unhelpful as the variance of the prior distribution is automatically increased with increasing expected fraction CSP. Properly setting the expected max CSP is critical for circumstances involving larger CSPs. Special care should be taken when ambiguous peak picking is possible. Setting the expected maximum CSP too large can allow the model to begin matching false peaks with distant peaks and lead to an unresolvable beam search. Fortunately, even under these circumstances, high confidence (>0.95) matches continue to yield reliable (>97%, accurate) matches. Lastly, execution on large data sets with hundreds of spectra is computationally feasible, all pHarmony runs in this study were executed in under 1 min with up to four runs operating in parallel on a 2023 Mac Book Pro (M3 Chip).

## Conclusions

A novel algorithm (pHarmony) for tracking peak movement due to a perturbation between two spectra has been described. pHarmony fills a gap left by existing tools: prior efforts emphasize automated peak picking (e.g., DEEP picker, UniDecNMR, CYPICK) or 2D-only CSP matching (e.g., PICASSO^4^), typically without formal confidence estimates or support for general multidimensional peak lists. The use of modern probabilistic machinery (mixture modeling, EM, SMC, beam search) is well aligned with current trends in quantitative NMR analysis. Cross-peak matching subject to random noise with and without chemical shift changes due to an external perturbation are well handled. While ligand binding was the perturbation tested here, the algorithm could be used for any kind of perturbation inducing peak movement (e.g., temperature, pH, salt, etc.). The algorithm maintains high accuracy in situations where cross-peaks may disappear either due to experimental phenomena (e.g., intermediate exchange) or data analysis errors (e.g., erroneous peak picking) and can work on peak lists of arbitrary dimensionalities. Importantly, the algorithm provides a robust confidence estimate of proposed matches that closely approximates true accuracy of the algorithm at high confidence levels. The algorithm has the advantage of being modular; it is possible to design more informative likelihood functions that consider other peak features e.g., (line width, intensity) in conjunction with distance that can then be sampled with SMC. The SMC decision space could also be expanded to account for two-to-one or one-to-two matches in the case of slow exchange. In addition, the Python package for pHarmony contains a peak matching function that can be imported into user-written Python scripts for seamless integration into automated workflows providing direct access to the estimated marginal matching distributions of the reference and target cross-peaks.

## Supplementary Material



## Data Availability

The Python package containing pHarmony is accessible through NMRbox and can be downloaded from https://github.com/anthony-bishop-tamu/pHarmony. pHarmony was written employing the NumPY,^25^ SciPY,^26^ Matplotlib,^27^ pandas^28^, PyTorch^29^, scikit-learn^30^, and tqdm^31^ Python packages as dependencies. The cross-peak lists for the analysis presented can be downloaded from the Texas Data Repository at 10.18738/T8/5MM8LJ.

## Notes

Figures of the flowchart of the pHarmony algorithm; influence of CSP priors; influence of maximum CSP. Comprehensive derivation of the pHarmony sequential Monte Carlo algorithm (PDF)
