# Supplementary material for: Harmonizing Peak Matching between Multidimensional NMR Spectra
Source: J Chem Inf Model. 2026 Aug 7;66(16):9961–75. doi: 10.1021/acs.jcim.6c00627 (PMC13508768; doi:10.1021/acs.jcim.6c00627)
Supplement: Supplementary file 1 [file ci6c00627_si_001.pdf]

Supporting Information

Harmonizing Peak Matching Between Multidimensional NMR Spectra

Anthony C. Bishop,[†]* Kyle Mimun,[†] Weimin Tan,[†] Taylor R. Cole[†] and A. Joshua Wand[†‡]*

Departments of Biochemistry & Biophysics[†] and Chemistry[‡]

Texas A&M University

College Station, Texas 77843 USA

Submitted to the Journal of Chemical Information and Modeling for consideration for publication as an Article.

* to whom correspondence should be addressed:
anthony.bishop@ag.tamu.edu; wand@tamu.edu

A. C. Bishop ORCID: 0000-0003-4853-1073

K. Mimun ORCID: 0000-0003-0060-4149

W. Tan ORCID: 0000-0002-6483-988X

T. R. Cole ORCID: 0000-0002-2025-5225

A. J. Wand ORCID: 0000-0001-8341-0782

SUPPORTING INFORMATION

SI Figures

SI Figure 1 – Flowchart of the pHarmony algorithm.

SI Figure 2 - The influence of the expected fraction CSP and variance scaling priors on pHarmony performance as a function of matching probability

SI Figure 3 - The influence of the expected maximum CSP on the performance of pHarmony.

Supporting Theory – Description of Sequential Monte Carlo (SMC) algorithm

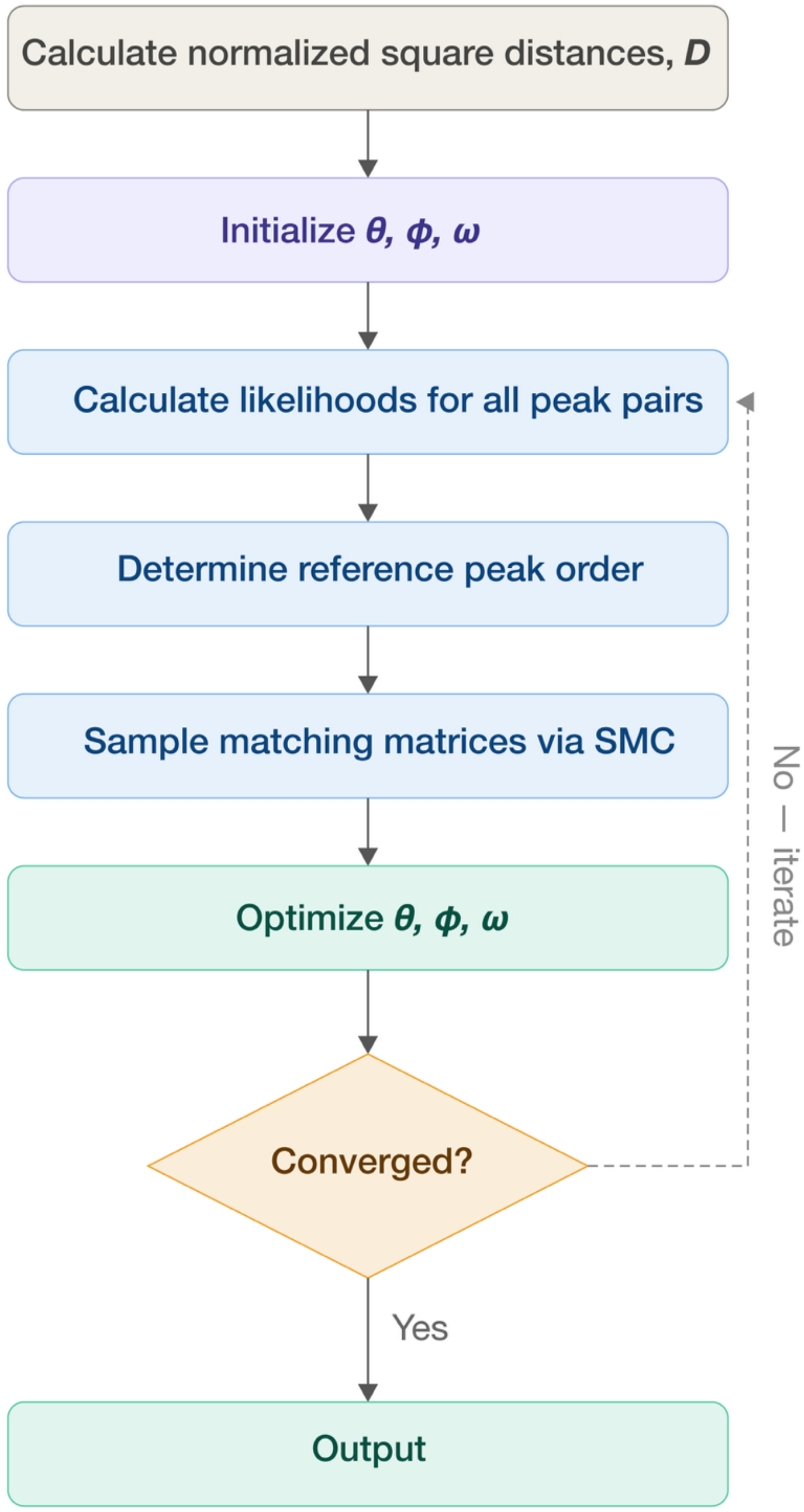

SI Fig 1 – Flowchart of the pHarmony algorithm.

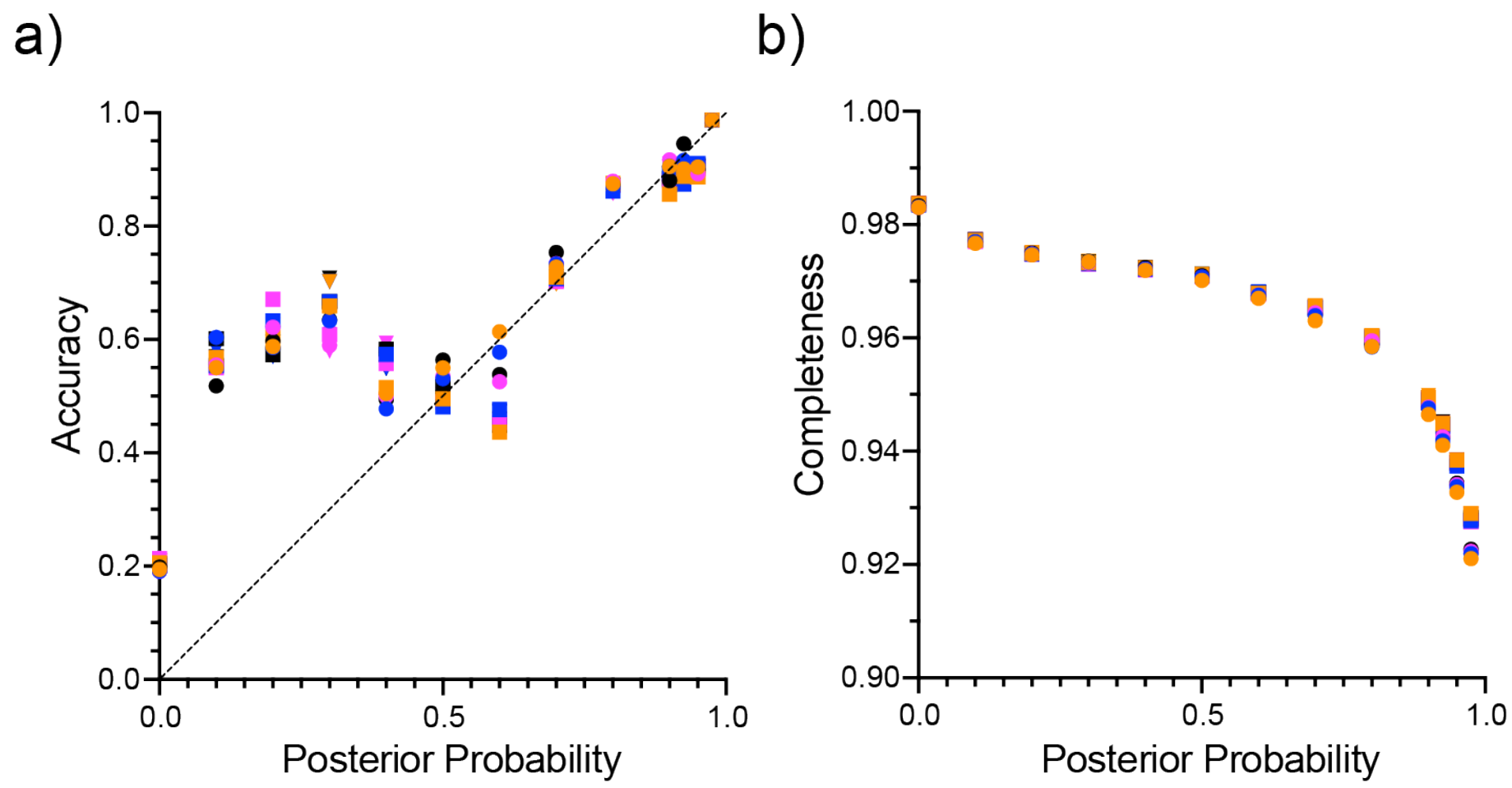

SI Fig. 2 – The influence of the expected fraction CSP and variance scaling priors on pHarmony performance as a function of matching probability (IL-1β HSQC data set, manually picked peaks). Panel A – accuracy, Panel B – completeness. The expected fraction CSP was set to either 0.001, 0.1 or 0.3 (circles, squares or triangles, respectively). The variance scaling factor was set to either 0.5, 1, 2 or 4 (orange, blue, magenta or black, respectively).

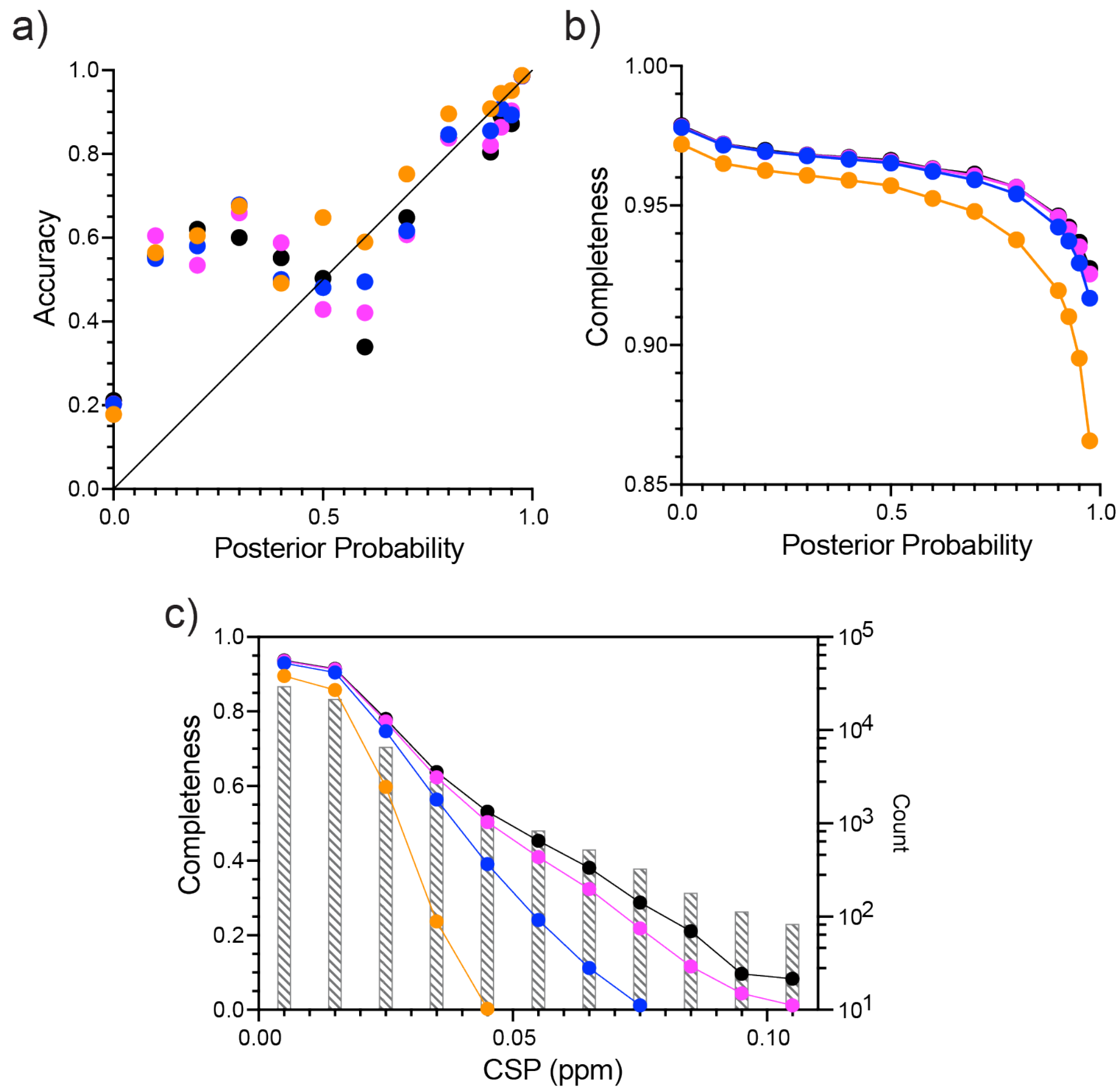

SI Fig. 3 – The influence of the expected maximum CSP on the performance of pHarmony. (IL-1β HSQC data set, manually picked peaks). Panel a – accuracy as a function of matching probability, Panel b – completeness as a function of matching probability. Panel c – histograms of detected and missed matches as a function of CSP magnitude for matches with a matching probability greater than 0.95. Matching was done with an expected maximum CSP of 0.05, 0.1, 0.15 or 0.2 ppm (orange, blue, magenta and black, respectively). Detected CSP histograms are indicated by solid lines. Missed CSPs are indicated by dashed lines.

Supporting Theory

Sampling the Posterior Distribution $\boldsymbol{P(M|\ D, \theta, \phi, \omega)}$. In the main text, (see Theory) a model is defined for describing the likelihood of any given matching matrix. Given a set of values θ, ϕ, ω that parameterize the likelihood function and the matrix *D,* we want to determine the posterior distribution of M. As noted in the main text, an exact evaluation of the distribution's pdf requires computing the likelihood of every possible matching matrix. To avoid this computationally prohibitive step, the matching matrix distribution is sampled using a sequential Monte Carlo sampler (SMC)[1], designed such that matching matrices are sampled with the probability described by equation 1 (main text). The sequential Monte Carlo sampler treats sampling a matching matrix as a series of decisions. First, a decision likelihood matrix (S) of dimensions R x $(T + 1)$ is constructed, where the likelihood at position *r, t* corresponds to the decision of matching reference peak *r* to target peak *t* and where position *r, T+1*, corresponds to the likelihood of reference peak *r* having no match in the dataset. The likelihood of the decision at position *r,t* (S_{rt}) is calculated by taking the product of the matching likelihood of peak *r* with target peak *t* with the product of all of the non-matching likelihoods with every other target peak. For the case of matching reference peak *r* with no target peak, the decision likelihood ($S_{r,T+1}$) is the product of the non-matching likelihoods of the reference peak with all target cross-peaks, i.e.,

$$S_{rt} = L(D_{rt}|\ M_{rt} = 1;\ \theta, \phi) \prod_{\tau\ \in T \backslash \{t\}} L(D_{r\tau}\ |\ M_{r\tau} = 0;\ \omega) \qquad \text{(SI 1)}$$

$$S_{r,T+1} = \prod_{t=1}^{T} L(D_{rt}\ |\ M_{rt} = 0;\ \omega) \qquad \text{(SI 2)}$$

The rationale behind calculating the decision likelihood in this manner is if reference peak *r* is matched to target peak *t*, then by the previously stated constraints *r* must also *not* match every other target peak. Similarly, the likelihood of not matching any target peak is the product of the non-matching likelihoods for each target peak.

Additionally, the constraints of our problem mean that the decision likelihood matrix itself will change as future decisions become unavailable. Once a decision is made for reference peak *r*, the likelihoods for all decisions in row *r* ($S_{r,*}$) are set to 0. In other words, once a decision for a row has been made, another decision cannot be made for that row. Likewise, if the decision S_{rt} is made, then the likelihood matrix for the entire column *t* ($S_{*,t}$) will be set to zero as a target peak cannot be matched twice. The exception is the column ($S_{*,T+1}$) which will always have a non-zero likelihood for all reference cross-peaks for which a decision has not yet been made (i.e., any number of reference cross-peaks can be labelled as not matching any target peak).

The SMC target and proposal distributions. Initially the SMC matching algorithm was implemented as described by Jun et al.[1]. However, it was determined that the simplified target distribution was insufficient to adequately match cross-peaks and following Jun et al., employed a proposal distribution that is calculated to match the target distribution equation 1 (main text) as closely as possible. Since each entry in the decision likelihood matrix describes the likelihood of an entire row of the matrix D_{rt} (i.e., each decision in row ($S_{r,*}$) represents a different possible $M_{r,*}$). The likelihood function $L(D \mid M_i;\ \theta, \phi, \omega)$ of the target distribution can be reparametrized as the product of a set of decisions:

$$L(D \mid \Sigma_i;\ \theta, \phi, \omega) = \prod_{\boldsymbol{r=1}}^{\boldsymbol{R}} L(D \mid \Sigma_{i,r};\ \theta, \phi, \omega) \qquad \text{(SI 3)}$$

Where Σ_i refers to any complete, valid decision set (i.e., a single decision made for each reference peak) and $\Sigma_{i,r}$ represents a valid decision for reference peak *r* and where *R* is the number of reference peaks. To emphasize, the likelihood function presented here is the same as defined earlier; the difference is the matching matrix is now represented by a sequence of decisions. This implies that any given Σ_i maps exactly one to one with a possible matching matrix M_i. We can then completely reparametrize equation 1 (main text) as:

$$P(\Sigma_i \mid D;\ \theta, \phi, \omega) = \frac{L(D \mid \Sigma_i\ ; \theta, \phi, \omega)}{Z} \qquad \text{(SI 4)}$$

$$Z = \sum_{j} L(D \mid \Sigma_j, \theta, \phi, \omega)$$

The summation over j involves summing the likelihoods of all possible complete decision sets. As before, the direct calculation of the partition function Z is intractable. Additionally, the prior probability terms have been dropped as we assume that all prior probabilities of all valid decision sets are equivalent to one another.

In SMC sampling, when drawing a sample of decision sets of size *N,* all members of the sample are drawn simultaneously. The probability of sample member *i* sampling decision set Σ_i is defined by a proposal probability $Q(\Sigma_i \mid D; \theta, \phi, \omega)$. Because the proposal distribution does not necessarily equal the target distribution, each member *i* in the sample is given an associated weight which is defined as the ratio of the target and proposal probabilities.

$$w_i = \frac{P\left(\Sigma^i \mid D; \theta, \phi, \omega\right)}{Q(\Sigma^i \mid D; \theta, \phi, \omega)} = \frac{L(D \mid \Sigma^i; \theta, \phi, \omega)}{Q(\Sigma^i \mid D; \theta, \phi, \omega)} \frac{1}{Z} \qquad \text{(SI 5)}$$

A new sample is drawn by randomly drawing members from the current sample (with replacement) with a probability proportional to their weight. The probability of drawing member *i* from the sample is equal to:

$$P(drawing\ member\ i) = \frac{w_i}{\sum_{j=1}^{N} w_j} = \frac{\frac{w_i{}'}{Z}}{\sum_{j=1}^{N} \frac{w_j{}'}{Z}} = \frac{w_i{}'}{\sum_{j=1}^{N} w_j{}'} \qquad \text{(SI 6)}$$

As the partition function *Z* has no bearing on the resampling probability, *Z* can be dropped from the calculation of the sample weights. Thus, weights can be calculated as the ratio of the likelihood of the target distribution to the proposal distribution probability.

Resampling guarantees that the drawn sample follows the target distribution. However, if the proposal distribution deviates significantly from the target, the weight can become concentrated in just a handful of sample members leading to high variance between samples. Because of this, it is helpful to resample before all decisions have been made. This allows sample members that have “fallen off track” and are exploring low probability

regions of the target distribution to be discarded. Resampling after a subset of decisions is possible given the ability to factorize our target distribution:

$$P(\Sigma_i \mid D;\ \theta, \phi, \omega) = \frac{\prod_{\boldsymbol{r=1}}^{\boldsymbol{R}} L(D \mid \Sigma_{i,r}; \theta, \phi, \omega)}{Z} \tag{SI 7}$$

Which can be rewritten as

$$P(\Sigma_i \mid D; \theta, \phi, \omega) = L(D \mid \Sigma_{i,1}; \theta, \phi, \omega) L(D \mid \Sigma_{i,2}; \theta, \phi, \omega) \ \dots\ L(D \mid \Sigma_{i,R}; \theta, \phi, \omega) \frac{\mathbf{1}}{Z} \tag{SI 8}$$

We can also define a target distribution over a set of partial matches

$$P\left(\Sigma_{i,1:R'} \mid D; \theta, \phi, \omega\right) = L(D \mid \Sigma_{i,1}; \theta, \phi, \omega) L(D \mid \Sigma_{i,2}; \theta, \phi, \omega) \dots L(D \mid \Sigma_{i,R'}; \theta, \phi, \omega) \frac{\mathbf{1}}{Z'} \tag{SI 9}$$

where $R' \le R$, $\Sigma_{1:R'}^{i}$ is the partial set of decisions over the first R' reference cross-peaks and Z' is the partition function over the decision set for the first R' reference cross-peaks. The proposal distribution can similarly be decomposed in the following manner.

$$Q\left(\Sigma_{i,1:R'} \mid D; \theta, \phi, \omega\right) = Q\left(\Sigma_{i,1} \mid D, \theta, \phi, \omega\right) Q\left(\Sigma_{i,2} \mid \Sigma_{i,1:1}, D; \theta, \phi, \omega\right) \dots Q\left(\Sigma_{i,R'} \mid \Sigma_{i,1:R'-1}, D; \theta, \phi, \omega\right) \tag{SI 10}$$

Each factor in the proposal distribution is essentially the proposal probability of the decision for each reference peak. It is important to note that the proposal probability has a conditional dependence that the target probability does not – i.e., the proposal probability distribution over the possible decisions of row *r* is dependent on all preceding decisions. This accounts for the fact that a target peak that was matched during a previous decision is no longer available for matching. Thus, the weight for sample member *i* after sampling decisions for the first R' rows is:

$$w_i = \frac{P\left(\Sigma_{i,1:R'} \mid D; \theta, \phi, \omega\right)}{Q\left(\Sigma_{i,1:R'} \mid D; \theta, \phi, \omega\right)} = \frac{L(D \mid \Sigma_{i,1:R'}; \theta, \phi, \omega)}{Q\left(\Sigma_{1:R'}^{i} \mid D; \theta, \phi, \omega\right)} \frac{1}{Z'} \tag{SI 11}$$

As before, the partition function Z' can be ignored thus permitting the weights to be calculated as the ratio of the target likelihood of the partial decision set to the proposal probability of the partial decision set. This enables accurate resampling at any step.

Computing the proposal probability. One possible proposal probability for the decision made at reference peak r is

$$Q\left(\Sigma_r^i \mid \Sigma_{1:r-1}; \theta, \phi, \omega\right) = \frac{L(D \mid \Sigma_r^i; \theta, \phi, \omega)}{\sum_{valid\ j} L(D \mid \Sigma_r^j; \theta, \phi, \omega)} = \frac{S_{ri}}{\sum_{valid\ j} S_{rj}} \qquad \text{(SI 12)}$$

Where the superscripts *i* and *j* index a possible decision available to reference peak r (i.e., that haven't been removed from consideration by earlier decisions). This proposal distribution however often leads to extremely uneven weights across the members of the sample and very high sample variance. The issue lies in the tendency to create seemingly good matches in the earlier decisions which preclude better matches that could have been made to later reference cross-peaks. Thus, a good proposal distribution needs to account for the effect of its current decision on the likelihood of all possible future decisions. The ideal proposal would be as follows:

$$Q\left(\Sigma_r^i \middle| \Sigma_{1:r-1}; \theta, \phi, \omega\right) = \frac{\sum_{all\ valid\ \Sigma_{r:R'}^i} L(D \mid \Sigma_{r:R'}^i; \theta, \phi, \omega)}{\sum_{valid\ j} \sum_{valid\ \Sigma_{r:R'}^j} L(D \mid \Sigma_{r:R'}^j; \theta, \phi, \omega)} \qquad \text{(SI 13)}$$

Where $\Sigma_{r:R'}^i$ refers to a partial set of decisions which matches reference peak r' with the target peak at index *i* (or the non-matching decision at *i = T+1*) and any possible decision for each of the subsequent rows up to row R'. Calculation of the exact proposal is intractable as the denominator of this equation, for the first decision ($r = 1, R' = R$), is the full partition function shown in equation 1 (main text). However, it is often the case that most of the sum over the possible likelihoods of partial decision sets $\Sigma_{r:R'}^i$ is contained in relatively few of the possible partial decision sets. Furthermore, a decision made at reference peak r will not substantially influence the probability of decisions made at all other reference cross-peaks, just a subset of them. Thus, if the reference cross-peaks that substantially affect each other's proposal distributions were ordered such that they were close in the

decision sequence, then the bulk of the *partial* partition function would be accurately estimated by considering only decisions at the "most relevant" subset of subsequent rows. In effect, the goal is to group the reference peaks in the decision sequence such that highly correlated reference peaks are close in sequence and to identify groups of reference peaks where decisions made for reference peaks in one group do not substantially affect the probabilities of decisions made for reference peaks in another group.

Reference Peak Ordering. Reference cross-peaks were ordered by first clustering the reference cross-peaks together via hierarchical clustering and then choosing an ordering that keeps the nearest neighboring cross-peaks close in sequence. A distance matrix for the purpose of clustering reference cross-peaks is calculated in the following way. First, for each reference peak, a subset of the target cross-peaks is chosen as matching candidates. Any target cross-peak with a matching likelihood that was less than the no match likelihood was eliminated from its candidate set. The no match decision was kept as a candidate for each reference cross-peak. The likelihoods of the candidate decisions were then normalized to a probability. A target peak was kept as a candidate if its probability was within a factor of 20 of the largest probability target peak for that reference cross-peak. This was done to eliminate target cross-peaks from a reference cross-peak's candidate set in cases where the target had a much higher probability of matching a different reference cross-peak. The number of overlapping candidate target cross-peaks between two reference cross-peaks was computed for all reference cross-peak pairs. For a common target cross-peak to be counted as overlap between two reference cross-peaks, the target must be present in both sets, and the ratio of the probability for the two reference-target cross-peak matches must not differ by more than a factor of 20. This ensures that overlap is only counted if there is a reasonable chance that the same target peak is matched to either reference peak. In other words, two reference cross-peaks can only be considered as "competing" for a target peak if the probability doesn't seem to overly favor one reference peak over the other.

After determining the number of overlapping candidates between each pair of reference cross-peaks, a distance between each pair of reference cross-peaks was calculated by

taking the multiplicative inverse of the number of overlapping target cross-peaks. Reference peak pairs with no overlap were assigned a distance of infinity. From this distance matrix, a weighted shortest path matrix was computed by finding the shortest path between all pairs of reference cross-peaks using the Floyd-Warshall algorithm[2]. For example, if the shortest path from reference peak A to C was via a third reference peak B, the path distance between A and C was set to the sum of the distance between A and B and the distance between B and C. The shortest path matrix was then used by the scikit-learn[3] AgglomerativeClustering class, using the single-linkage clustering algorithm and a distance threshold of 1.0. This guarantees any two reference cross-peaks that have a path of non-infinite distance between them will belong to the same cluster. Using the clustering results, a linkage matrix defining a dendrogram was calculated which was passed to the scipy[4] optimal leaf ordering algorithm, the results of which became the decision order for the reference cross-peaks. This resulted in a reference peak order with independent clusters of reference peaks, where highly correlated reference peaks were placed proximally in the decision sequence within a cluster.

Beam Search. To compute the proposal distribution a beam search strategy[5] was used to estimate the partition function over the remaining decisions within a cluster of reference peaks. For reference cross-peak r a beam search of width 1000 is performed over all of the remaining reference cross-peaks in that cluster. From a given candidate decision within r, a beam is sent to every candidate at reference $r+1$. The beams then split such that each $r+1$ candidate sends a beam to each candidate decision at $r+2$. With the repeated branching the number of beams grows exponentially. Should the number of beams exceed 1000 at any time only the 1000 beams with the highest likelihoods are retained, with the likelihood of each beam being the product of the likelihoods of the decisions it visits. This continues until the last reference peak in the cluster is reached. The likelihoods of all of the beams are then summed together, providing the proposal weights for that decision at reference peak r. This is repeated for each possible decision in r. The weights for each decision are normalized over the decisions to yield the proposal probability distribution for the decisions of reference peak r.

Resampling and sample size determination. Once a decision for the last reference peak in a cluster has been made, the effective sample size (ESS) ratio (defined as the ratio of the effective sample size to the sample size *N*) is calculated.

$$ESS\ Ratio = \frac{\left(\sum_{i=1}^{N} w_i\right)^2}{N \sum_{i=1}^{N} {w_i}^2} \qquad \text{(SI 14)}$$

The ESS ratio is indicative of how well the proposal distribution matched the target distribution across the sampled decisions with a value of 1.0 indicative of perfect agreement and 0 being very poor agreement. If an ESS ratio of 0.2 or larger is measured the sample is resampled by stratified resampling[6] where a new sample is drawn from the current sample (with replacement) such that the probability of drawing a member of the sample is proportional to its sample weight. If the ESS ratio is less than 0.2, indicative of poor agreement between proposal and target distributions, all decisions made on the reference peaks in the current cluster are discarded and the algorithm returns to the first cross peak in the cluster. The maximum width of the beam search is increased by a factor of five and the algorithm proceeds as normal. This process repeats with progressively increasing the maximum beam size until the local partition function is sufficiently estimated to generate an ESS ratio of 0.2 or greater. If the maximum beam search width becomes so large such that an internal memory threshold is exceeded, the program errors out. This occurrence seems quite rare in practice and has only been seen in cases of highly unrealistic peak lists (e.g. grossly incorrect peak picking).

At the beginning of the algorithm, sample size *N* is initialized to the number of reference cross-peaks. After sampling the posterior distribution, the frequency of each reference-target cross-peak match is calculated, and a second sample is drawn. The frequencies of each reference-target match are compared. If the difference in frequency for any reference-target pair is greater than 0.1, *N* is increased by a factor of two and the sampling is repeated.

References

(1) Jun, S. H.; Wong, S. W. K.; Zidek, J. V.; Bouchard-Côté, A. Sequential Graph Matching with Sequential Monte Carlo. *J. Mach. Learning Res.* **2017**, *54*, 1075-1084.

(2) Floyd, R. W. Algorithm 97: Shortest path. *Commun ACM* **1962**, *5* (6), 345-345. DOI: 10.1145/367766.368168.

(3) Pedregosa, F.; Varoquaux, G.; Gramfort, A.; Michel, V.; Thirion, B.; Grisel, O.; Blondel, M.; Prettenhofer, P.; Weiss, R.; Dubourg, V.; et al. Scikit-learn: Machine Learning in Python. *J. Mach. Learning Res.* **2011**, *12*, 2825-2830.

(4) Virtanen, P.; Gommers, R.; Oliphant, T. E.; Haberland, M.; Reddy, T.; Cournapeau, D.; Burovski, E.; Peterson, P.; Weckesser, W.; Bright, J.; et al. SciPy 1.0: fundamental algorithms for scientific computing in Python. *Nat. Methods* **2020**, *17* (3), 261-272. DOI: 10.1038/s41592-019-0686-2.

(5) Ow, P. S.; Morton, T. E. Filtered beam search in scheduling. *Int. J. Prod. Res.* **1988**, *26* (1), 35-62. DOI: 10.1080/00207548808947840.

(6) Kitagawa, G. Monte Carlo Filter and Smoother for Non-Gaussian Nonlinear State Space Models. *J. Comp. Graph. Stat.* **1996**, *5* (1), 1-25. DOI: 10.1080/10618600.1996.10474692.
